# Early activation of inflammatory pathways in *UBA1-*mutated hematopoietic stem and progenitor cells in VEXAS

**DOI:** 10.1016/j.xcrm.2023.101160

**Published:** 2023-08-15

**Authors:** Zhijie Wu, Shouguo Gao, Qingyan Gao, Bhavisha A. Patel, Emma M. Groarke, Xingmin Feng, Ash Lee Manley, Haoran Li, Daniela Ospina Cardona, Sachiko Kajigaya, Lemlem Alemu, Diego Quinones Raffo, Amanda K. Ombrello, Marcela A. Ferrada, Peter C. Grayson, Katherine R. Calvo, Daniel L. Kastner, David B. Beck, Neal S. Young

**Affiliations:** 1 Hematology Branch, National Heart, Lung, and Blood Institute, National Institutes of Health, Bethesda, MD 20892, USA; 2 National Human Genome Research Institute, National Institutes of Health, Bethesda, MD 20892, USA; 3 National Institute of Arthritis and Musculoskeletal and Skin Diseases, National Institutes of Health, Bethesda, MD 20892, USA; 4 Hematology Section, Department of Laboratory Medicine, National Institutes of Health, Bethesda, MD 20892, USA; 5 Division of Rheumatology, Department of Medicine, New York University Grossman School of Medicine, New York, NY 10016, USA; 6 Center for Human Genetics and Genomics, New York University Grossman School of Medicine, New York, NY 10016, USA

**Keywords:** VEXAS, single-cell RNA sequencing, inflammation, clonal hematopoiesis

## Abstract

VEXAS (vacuoles, E1 enzyme, X-linked, autoinflammatory, somatic) syndrome is a pleiotropic, severe autoinflammatory disease caused by somatic mutations in the ubiquitin-like modifier activating enzyme 1 (*UBA1*) gene. To elucidate VEXAS pathophysiology, we performed transcriptome sequencing of single bone marrow mononuclear cells and hematopoietic stem and progenitor cells (HSPCs) from VEXAS patients. HSPCs are biased toward myeloid (granulocytic) differentiation, and against lymphoid differentiation in VEXAS. Activation of multiple inflammatory pathways (interferons and tumor necrosis factor alpha) occurs ontogenically early in primitive hematopoietic cells and particularly in the myeloid lineage in VEXAS, and inflammation is prominent in *UBA1*-mutated cells. Dysregulation in protein degradation likely leads to higher stress response in VEXAS HSPCs, which positively correlates with inflammation. TCR usage is restricted and there are increased cytotoxicity and IFN-γ signaling in T cells. In VEXAS syndrome, both aberrant inflammation and myeloid predominance appear intrinsic to hematopoietic stem cells mutated in *UBA1*.

## Highlights

• Inflammation and myeloid dominance originate in hematopoietic stem cells in VEXAS syndrome
• mt*UBA1* myeloid cells upregulate inflammatory pathways compared with wild-type cells
• There are biased granulocytic differentiation and increased proliferation of mt*UBA1* cells
• Apoptosis of mt*UBA1* lymphoid progenitors causes impaired lymphocytopoiesis in disease

## Graphical abstract

**Figure:**
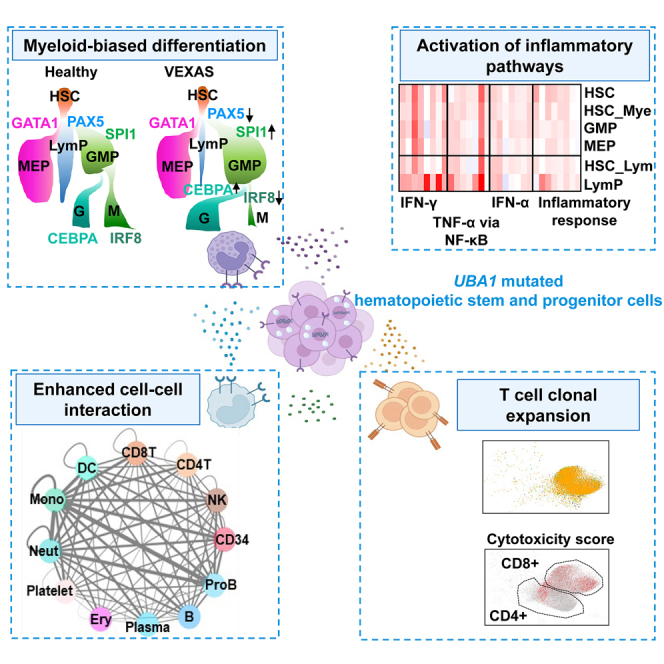

## Teaser

To address the link between *UBA1* mutations and hyperinflammation in VEXAS syndrome, Wu et al. present single-cell transcriptome analyses on bone marrow of VEXAS patients. Myeloid lineage bias and inflammatory gene activation occur early in hematopoietic stem cells and appear intrinsic to *UBA1* mutant cells highlighting mechanism of clonal dominance.

## Introduction

VEXAS (vacuoles, E1 enzyme, X-linked, autoinflammatory, somatic) syndrome is a recently identified inflammatory disease caused by somatic mutations in *UBA1*, an X chromosome gene encoding the ubiquitin-like modifier-activating enzyme 1 (UBA1).^1^ VEXAS is an example of an emerging class of disorders with overlapping rheumatologic and hematologic manifestations caused by acquired mutations. VEXAS in particular may be relatively frequent, as the mutation has been identified in about 1 in 4,269 men older than 50 years.^2^ VEXAS features cytopenias, bone marrow (BM) dysplasia, and striking vacuolization of BM precursors, and patients have fever and a variety of organ-specific inflammatory manifestations.^1^^,^^3^^,^^4^^,^^5^^,^^6^^,^^7^ Following our initial report, *UBA1* mutations have been discovered in such common clinical diagnoses as giant cell arteritis, relapsing polychondritis, polyarteritis nodosa, Sweet syndrome, and myelodysplastic syndrome (MDS).^3^^,^^4^^,^^5^^,^^6^^,^^7^^,^^8^^,^^9^^,^^10^^,^^11^^,^^12^^,^^13^^,^^14^^,^^15^^,^^16^^,^^17^^,^^18^^,^^19^^,^^20^^,^^21^ However, the pathophysiology of VEXAS remains unclear, especially the relationship between a genetic defect in a hematopoietic stem cell and such diverse abnormalities of immunologic signaling, BM failure, myeloid neoplasm, and plasma cell dyscrasias.

Acquired mutations are age related and are originally implicated in cancer; they have now been recognized as key drivers of “benign” diseases across medical subspecialties.^22^ Benign (in the sense of not cancer) somatic mutation diseases are familiar to hematologists: paroxysmal nocturnal hemoglobinuria, a thalassemia-like syndrome in MDS, and histiocytoses are examples. Somatic mutations may also contribute to other complex multifactorial processes such as atherosclerosis and clonal hematopoiesis of indeterminate potential (CHIP).^23^

VEXAS is an example of a non-malignant disease secondary to acquired mutations. *UBA1* “knockout” causes inflammation in zebrafish, but animal models of VEXAS are limited by germline, rather than somatic, loss of *UBA1*,^1^ and VEXAS cells are unusually fragile, intolerant of minimal *in vitro* manipulation, and poorly proliferative in culture. For these reasons, it has remained unclear how a specific genetic defect in hematopoietic cells results in refractory inflammation, marrow failure with dysplasia, and plasma cell dyscrasias. Alternatively, analogous to somatic mutations that are frequent in hematopoietic diseases, positive selection of clones containing somatic mutations by environmental factors might drive clonal dominance of mutated cells.^21^ Identification of differences between wild-type and mutant cell populations would be useful to understand pathologic mechanisms and to select and design treatments. Due to absent cellular and animal models, direct observation of patient cells is both advantageous and immediately relevant to elucidating the pathogenesis of VEXAS.

Single-cell genomic methods are appropriate to a disease like VEXAS: they require little sample manipulation, avoid culture artifacts, and allow for detection of rare cell types. Associated computational analyses are largely free of *a priori* bias in utilizing open-ended approaches to data collection and processing. Single-cell studies can directly detect genotypes and transcriptomes within cells and disclose altered pathways involved in disease.^24^^,^^25^^,^^26^^,^^27^^,^^28^^,^^29^^,^^30^^,^^31^^,^^32^^,^^33^^,^^34^

Here, we performed single-cell RNA sequencing (scRNA-seq) and single-cell T cell receptor/B cell receptor sequencing (scTCR/BCR-seq) of BM mononuclear cells (BMMNCs) and enriched hematopoietic stem and progenitor cells (HSPCs) from nine patients included in the original VEXAS report^1^ as an exploratory cohort. Myeloid dominance and inflammation (especially, tumor necrosis factor alpha [TNF-α] and interferon gamma [IFN-γ]) originated early in lineage-restricted progenitors and myeloid precursors in VEXAS. These findings were then validated in an independent cohort of patients using functional immunologic assays*. UBA1*-mutated (mt*UBA1*) myeloid cells exhibited upregulated inflammatory pathways and immune activation compared with wild-type *UBA1* (wt*UBA1*). We implicate lineage bias toward myeloid, and granulocytic differentiation in particular, intrinsic increased cell cycling of mt*UBA1* myeloid cells, and increased apoptosis of mt*UBA1* lymphoid progenitors (LymPs) in clonal dominance of myeloid cells and loss of lymphocyte populations, respectively. Dysregulated protein degradation and therefore increased stress response were observed, and stress responses positively correlated with the VEXAS inflammatory signature. We also profiled cell-cell interactions of marrow myeloid cells with HSPCs and TCR and BCR repertoires in VEXAS. Our work presents detailed description of single hematopoietic cells in VEXAS, and our results should facilitate our understanding of hematopoiesis, clonal dominance, cell-cell interactions, and TCR/BCR repertoires in this newly defined syndrome. Furthermore, they suggest potential utility of TNF and IFN blockades in the treatment of VEXAS syndrome.

## Results

### Patient characteristics

**Figure 1:**
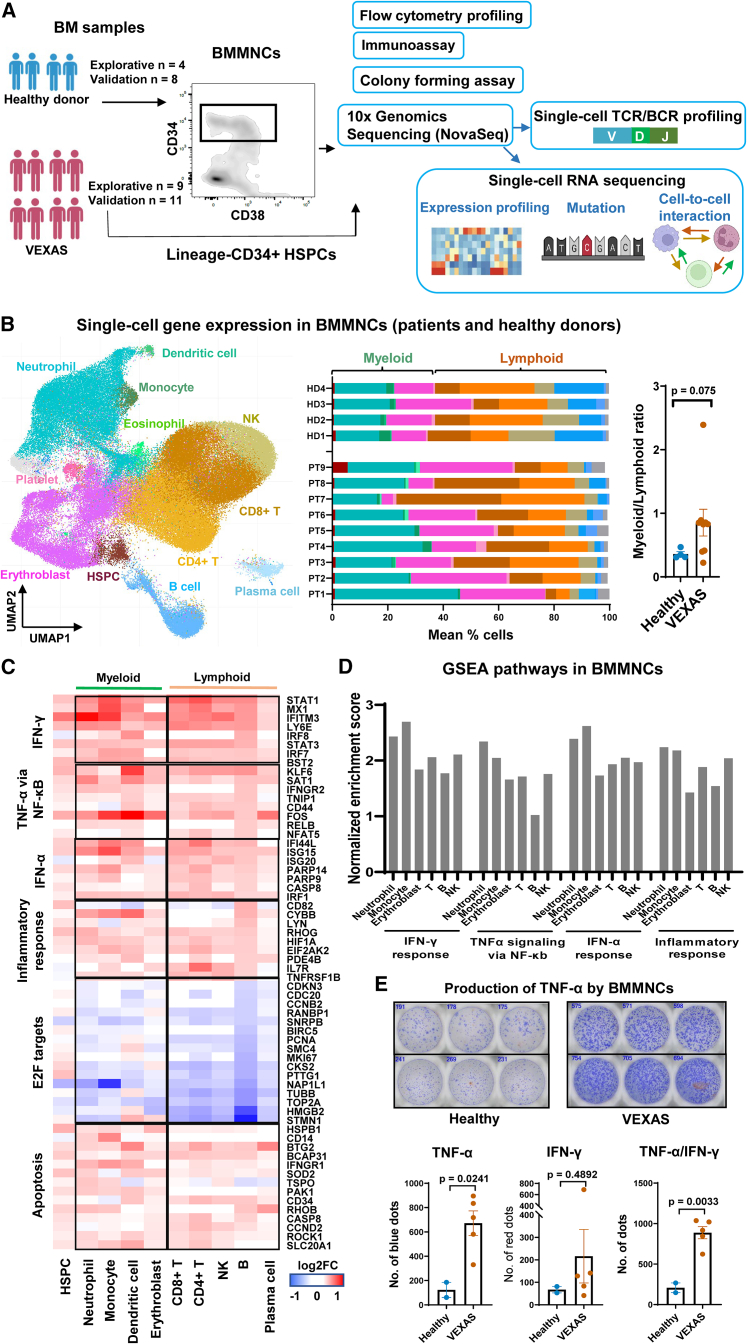
Myeloid dominance and activation of the inflammatory pathways in VEXAS BMMNCs (A) Experimental workflow. BMMNC samples from patients and healthy donors were subjected to multi-color flow cytometry to profile hematopoietic stem and progenitor cell (HSPC) subpopulations, and to ELISpot assay to quantify BMMNCs secreting TNF-α or IFN-γ. BMMNCs and FACS-sorted Lineage^−^CD34^+^ cells were subjected to colony forming assay and single-cell RNA sequencing (scRNA-seq) using the 10x Genomics platform. scRNA-seq libraries were sequenced on the Illumina NovaSeq system before data analysis, including single-cell transcriptome profiling (gene expression, gene mutation, and cell-cell interaction) and single-cell T cell receptor/B cell receptor (scTCR/BCR) profiling. (B) A Uniform Manifold Approximation and Projection (UMAP) plot of single-cell gene expression in BMMNCs of all patients and healthy donors. Cells are colored by types (HSPC, erythroblast, neutrophil, monocyte, T cell, NK cell, B cell, plasma cell, eosinophil, and dendritic cell). A bar chart shows percentages of these cell populations in individual patients and healthy donors. The color legend is the same as that in the UMAP plot. A dot plot showing a myeloid (erythroblast, neutrophil, monocyte, and dendritic cell) vs. lymphoid (T cell, B cell, NK cell, and plasma cell) ratio in patients and healthy donors. Data are presented as mean values ± standard error of the mean (SEM). p values with the two-sided unpaired Mann-Whitney test are shown. (C) Heatmap showing expression of representative differentially expressed genes grouped by their functional pathways in IFN-γ and IFN-α signaling, TNF-α via NF-κB signaling, inflammatory response, E2F targets, and apoptosis, between BMMNCs from VEXAS patients (n = 9) and healthy controls (n = 4). Values are presented as log2 fold-changes (log2FC). (D) Gene set enrichment analysis (GSEA) of expressed genes in BMMNC subpopulations of VEXAS patients, including neutrophils, monocytes, erythroblasts, T cells, B cells, and NK cells. Normalized enrichment scores for the GSEA pathways are plotted, showing higher enrichment of the inflammatory pathways in neutrophils and monocytes than those in lymphoid cells. (E) Representative ELISpot wells showing TNF-α secretion by BMMNCs from two VEXAS patients and two healthy donors in a second batch of the validation cohort, in triplicate. Bottom, quantification of TNF-α-, IFN-γ-, and TNF-α/IFN-γ-positive spots in BMMNCs plated (VEXAS patients n = 5 and healthy donors n = 2, in triplicate). Data are presented as mean values ± standard error of the mean (SEM). p values with the two-sided unpaired Mann-Whitney test are shown.

An exploratory cohort composed of nine patients (all males and median age 65 years, and reported earlier) with confirmed *UBA1* mutations underwent scRNA-seq, scTCR/BCR-seq, flow cytometry profiling, and colony formation. For validation, we enrolled another 11 patients (all males and medium age 67 years) for immunoassays and flow cytometry profiling (Figure 1A). Patient clinical characteristics are summarized in Table S1.

### A distinct transcriptional profile of BM cells in VEXAS

We first sought to study gene expression of hematopoietic cells using scRNA-seq. After quality control, 84,401 single BMMNCs from nine patients and 36,425 from four healthy individuals were retained for further analyses.

Sequences from single BMMNCs were visualized in Uniform Manifold Approximation and Projection (UMAP). Cells formed clusters, imputed from similarity among transcriptomes with a graph-based approach,^35^ from which BMMNC subpopulations could be assigned computationally: CD34^+^ HSPCs, erythroblasts, neutrophils (a sum of neutrophil lineages at different differentiation stages), monocytes (a sum of monocytic lineages at different differentiation stages), CD4^+^ T cells, CD8^+^ T cells, B cells, plasma cells, natural killer (NK) cells, dendritic cells, and platelets (Figures 1B and S1A). By deconvoluting single cells of a heterogeneous BMMNC population, we determined that patients had increased myeloid cell (particularly granulocyte) proportions in their marrows (Figure 1B).

Gene expression of BMMNCs in patients was compared with that in healthy individuals: many genes were differentially expressed in VEXAS. By gene ontology (GO) analysis, aberrantly expressed genes were mainly involved in functions related to the immune response, leukocyte activation, cell communication, and protein metabolism (Figure S1B). IFN signaling, TNF-α signaling, and inflammatory response were upregulated in various cell types (Figures 1C and S1C) but predominately in myeloid cells (Figure 1D). These data were consistent with transcriptome changes described for peripheral blood cells.^1^ Cell cycling genes (e.g., E2F targets) were downregulated while apoptosis genes were upregulated in VEXAS. In an independent validation cohort (11 VEXAS patients and 8 healthy donors), we confirmed heterogeneous patterns of abundant TNF-α and IFN-γ secretion by VEXAS BMMNCs (Figures 1E and S1D).

### Myeloid bias and activation of the inflammation pathways in VEXAS HSPCs

**Figure 2:**
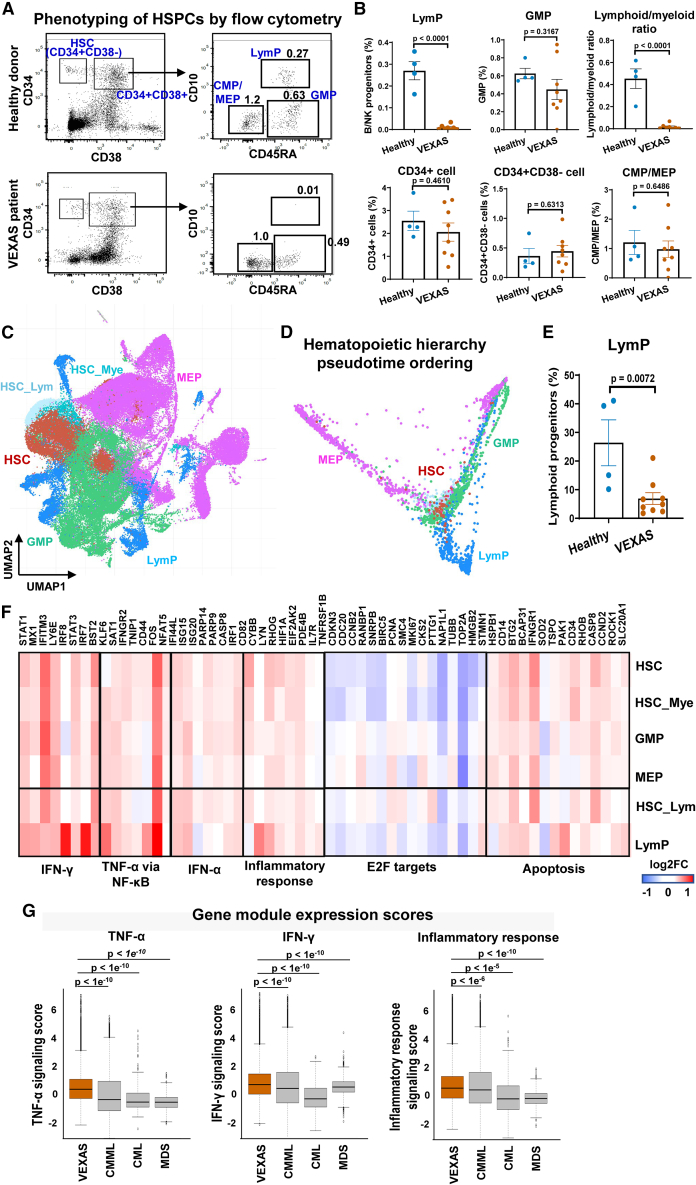
Myeloid bias and activation of the inflammatory pathways in VEXAS HSPCs (A) Phenotypes of HSPCs in healthy donors and VEXAS patients by flow cytometry. Cell populations were defined as reported^36^: HSC, Lineage^−^CD34^+^CD38^−^; CMP/MEP, Lineage^−^CD34^+^CD38^+^CD10^−^CD45RA^−^; GMP, Lineage^−^CD34^+^CD38^+^CD10^−^CD45RA^+^; LymP, Lineage^−^CD34^+^CD38^+^CD10^+^; HSC, hematopoietic stem cells and multipotent progenitors; CMP, multipotent common myeloid progenitor; MEP, megakaryocytic-erythrocytic progenitors; GMP, granulocytic-monocytic progenitors; LymP, lymphoid progenitors. (B) Proportions of progenitor populations were compared between VEXAS patients (n = 9) and healthy donors (n = 4). Data are shown with mean values ± SEM. p values with the two-sided unpaired Mann-Whitney test are shown. (C) A UMAP plot of single-cell gene expression in HSPCs of all patients and healthy donors. Cells are colored by cell types as HSC, MEP, GMP, LymP, HSC_Lym (HSC with lymphoid differentiation potential), and HSC_Mye (HSC with myeloid differentiation potential). (D) Reconstruction of hematopoietic hierarchy pseudotime ordering with Palantir. The color legend is the same as in (C). (E) Percentages of LymPs were compared between VEXAS patients (n = 9) and healthy donors (n = 4). Data are presented as mean values ± SEM. p values with the two-sided unpaired Mann-Whitney test are shown. (F) Heatmap showing expression of representative differentially expressed genes grouped by their functional pathways in IFN-γ and IFN-α signaling, TNF-α via NF-κB signaling, inflammatory response, E2F targets, and apoptosis, between HSPCs from VEXAS patients (n = 9) and healthy controls (n = 4). Values are presented as log2FC. (G) Relative inflammatory pathway scores (TNF-α signaling, IFN-γ signaling, and inflammatory response signaling scores) in VEXAS, CMML, CML, and MDS patients.^37^^,^^38^^,^^39^ p values with the two-sided unpaired t test are shown.

To characterize early hematopoiesis in VEXAS, flow cytometry using an established panel of cell surface markers^36^ was performed to profile BM stem cells and progenitors. The numbers of CD34^+^ HSPCs, CD34^+^CD38^−^ stem cells, and multipotent progenitor cells were not different between patients and controls, but there was a marked reduction in LymP numbers and thus a significantly decreased lymphoid/myeloid cell progenitor ratio in VEXAS samples (Figures 2A and 2B), consistent with myeloid cell dominance in BM and peripheral blood in this disease.^1^^,^^3^ This result was confirmed in an independent cohort of patients (Figures S2A and S2B).

We next queried whether myeloid dominance and activation of inflammatory gene programs originated from HSPCs. We examined transcriptomes of enriched Lineage^−^CD34^+^ HSPCs by scRNA-seq; after quality control, 62,103 single HSPCs from patients and 52,272 from healthy individuals were retained for further analyses. From published cell type signatures,^40^ we deconvoluted single cells as stem cells and multipotent progenitors (HSCs), megakaryocyte-erythrocyte progenitors (MEPs), granulocyte-monocytic progenitors (GMPs), and LymPs (Figures 2C and S2C). When the hematopoietic hierarchy was reconstructed by pseudotemporal ordering, we observed the anticipated three major differentiation trajectories: from HSCs to MEPs, to myeloid cells, and to lymphoid cells (Figure 2D). Similar to flow cytometric phenotyping, there were markedly reduced LymPs in all VEXAS patients (Figure 2E).

Many differentially expressed genes were identified based on gene expression in stem cells and lineage-committed progenitors from patients with VEXAS syndrome. Gene set enrichment analysis was employed to characterize skewed gene sets. Genes involved in the immune response mediated by IFN-α, IFN-γ, and TNF-α, and the general inflammatory response, observed to be upregulated in VEXAS BMMNCs, were highly enriched in patients’ HSPCs (Figures S2D and S2F). Similar to BMMNCs, cell cycling genes (e.g., E2F targets) were downregulated and apoptosis genes were upregulated in HSPCs. From these striking changes, we inferred global effects of *UBA1* mutations on early hematopoiesis, resulting in myeloid dominance and inflammatory gene pathway activation.

VEXAS syndrome shares overlapping clinical features with other hematologic diseases (such as MDS, chronic myelomonocytic leukemia [CMML], and chronic myeloid leukemia [CML]), including BM proliferation and dysplasia, myeloid cell dominance, and frequent co-occurrence of inflammatory or autoimmune disorders. CMML features Sweet syndrome and other inflammatory manifestations, and MDS has historically been associated with inflammation and frank autoimmunity in various organs.^41^^,^^42^^,^^43^^,^^44^^,^^45^^,^^46^^,^^47^ To investigate whether inflammation observed in early HSPCs was present in these other diseases, we integrated our scRNA-seq data from sorted Lineage^−^CD34^+^ cells with published data of HSPCs in MDS, CMML, and CML patients.^37^^,^^38^^,^^39^ We batch-corrected and integrated across samples, and then compared relative expression levels of the inflammatory pathways (activity scores of the IFN-γ response, TNF-α response, and inflammatory response pathways) in VEXAS patients with those in patients with the other three diseases; inflammation was indeed present in these myeloproliferative and MDSs but more extreme in VEXAS (Figure 2G).

### mt*UBA1* HSPCs exhibit active cell cycling and increased inflammatory gene expression

Within the scRNA-seq data, we were able to identify mt*UBA1* single cells from their mRNA sequences. Due to “dropout” and other limitations of the platform, mt*UBA1* transcripts were only captured in a fraction of total cells (∼9% of BMMNCs and 20% of CD34^+^ HSPCs) and preferentially in some patients (better in UPNs 14–17 [with 5′ capture] than in UPNs 1, 6, 10, 11, and 13 [with 3′ capture]). Captured *UBA1* mutations were identical to concomitant Sanger sequences of bulk samples (Figure S3A). *UBA1* transcripts were detected more readily in CD34^+^ cells than in BMMNCs, presumably due to higher *UBA1* mRNA expression early in hematopoietic ontogeny (Figures S3B and S3C); there was higher frequency of mt*UBA1* transcripts in CD34^+^ cells than in BMMNCs (Table S2), consistent with digital PCR data in our original report.^1^ As the *UBA1* gene is located on the X chromosome and all patients were male, there is only a single *UBA1* allele; we denoted single cells containing at least one mt*UBA1* mRNA transcript as mt*UBA1* cells, single cells with only wt*UBA1* mRNA transcripts as wt*UBA1* cells, and single cells with no *UBA1* mRNA transcripts as “unknown.”^48^

**Figure 3:**
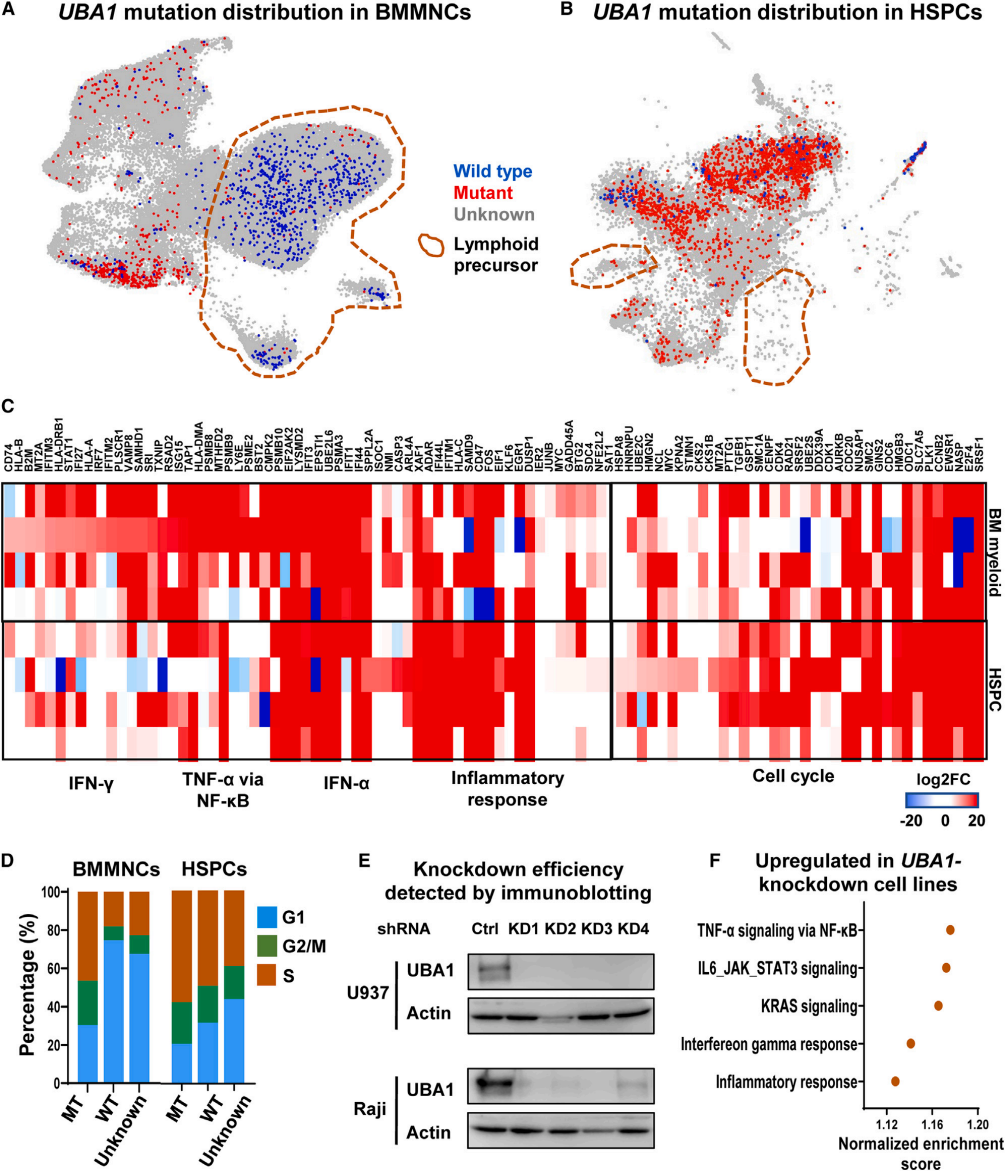
*UBA1*-mutated HSPCs exhibit increased inflammation and active cell cycling (A) A UMAP plot of single-cell gene expression in BMMNCs of VEXAS patients, as in Figure 1B. Cells with expressed mutated *UBA1* (mt*UBA1*) and wild-type *UBA1* (wt*UBA1*) are colored red and blue, respectively, and all the other cells in gray. Lymphoid precursors are circled on the UMAP plot. (B) A UMAP plot of HSPCs of VEXAS patients, the same as Figure 2C. Cells with expressed mt*UBA1* and wt*UBA1* are colored red and blue, respectively, and all the other cells in gray. (C) A heatmap showing expression of representative differentially expressed genes grouped by their functional pathways in IFN-γ and IFN-α signaling, TNF-α via NF-κB signaling, inflammatory response, and cell cycling, between mt*UBA1* and wt*UBA1* BMMNCs (top) and HSPCs (bottom) in VEXAS patients (n = 9). Values are presented as log2FC. (D) Bar plots showing percentages of BMMNCs (left) and HSPCs (right) in G1, G2/M, and S phases of cell cycle in mt*UBA1*, wt*UBA1*, and NULL cells in VEXAS patients. (E) Immunoblotting results showing knockdown efficiency of *UBA1* in cell lines (U937 and Raji). (F) A dot plot showing top GO terms enriched in upregulated genes in *UBA1* knockdown cell lines (U937, THP1, Raji, and Jurkat) compared with those in wild-type control cell lines.

To correlate *UBA1* mutations with transcription, we first overlaid mutation data on a UMAP plot by highlighting mt*UBA1* and wt*UBA1* cells, assuming that *UBA1* was expressed ubiquitously, and therefore that nonuniform distribution of mt*UBA1* cells on UMAP would indicate distinct contributions of gene mutations to gene expression. mt*UBA1* cells were most enriched in myeloid cells of BMMNCs and myeloid progenitors in HSPCs (Figures 3A and 3B), suggesting contributions of *UBA1* mutations to myeloid dominance. We compared mt*UBA1* and wt*UBA1* myeloid cell gene expression to stratify potential effects of cell type distribution. Genes involved in the inflammatory pathways were upregulated in both mt*UBA1* BMMNCs and mt*UBA1* HSPCs; cell cycling genes also were upregulated in mt*UBA1* cells (Figures 3C, S3D, and S3E; Table S2). A higher proportion of mt*UBA1* cells were in S phase than were wt*UB*A1 (and unknown cells; Figure 3D). In summary, from a comparison between mt*UBA1* cells with wt*UBA1* cells, we inferred *UBA1* mutations linked to immune activation, myeloid dominance, active cell cycling in stem cells and early progenitor cells, and myeloid lineage precursors in VEXAS BM.

To obtain direct evidence that loss of wt*UBA1* resulted in major alterations in transcription, we “knocked down” *in vitro UBA1* expression in two myeloid (U937 and THP1) and two lymphoid (Raji and Jurkat) cell lines (Figures 3E and S4A). Expression of inflammation genes, including genes involved in the TNF-α and IFN-γ pathways, increased in all four perturbed myeloid and lymphoid cell lines (Figure 3F; Table S3). These results supported a cell-autonomous mechanism of hyperinflammation due to deficiency of wt*UBA1*.

*DNMT3A* somatic mutations are frequent in myeloid neoplasms such as acute myeloid leukemia and MDS, and they are observed at unusually high frequency in VEXAS.^18^^,^^19^^,^^20^^,^^21^ UPN1 and UPN5 had *DNMT3A* mutations at variant allele frequency 40%–50%. To understand *DNMT3A*-mutated (mt*DNMT3A*) clones in the context of VEXAS syndrome, we identified single cells expressing *DNMT3A* mutations from mRNA sequencing (Figure S4B). As there are two alleles of the *DNMT3A* gene, we denoted single cells with at least one mt*DNMT3A* mRNA transcript as mt*DNMT3A* cells, single cells with only wild-type *DNMT3A* (wt*DNMT3A*) mRNA transcripts as wt-likely *DNMT3A* cells, and single cells with no *DNMT3A* mRNA transcripts as unknown.^48^ As for *UBA1* mutations, expressed *DNMT3A* mutations were mainly in myeloid cells (Figures S4C–S4F). In both BMMNCs and HSPCs, the immune response- and inflammation-related pathways were upregulated, but the cell-cycle-related pathways were downregulated in mt*DNMT3A* cells compared with wt-likely *DNMT3A* cells (Table S4).

### Perturbed protein degradation and unfolded protein response in VEXAS

**Figure 4:**
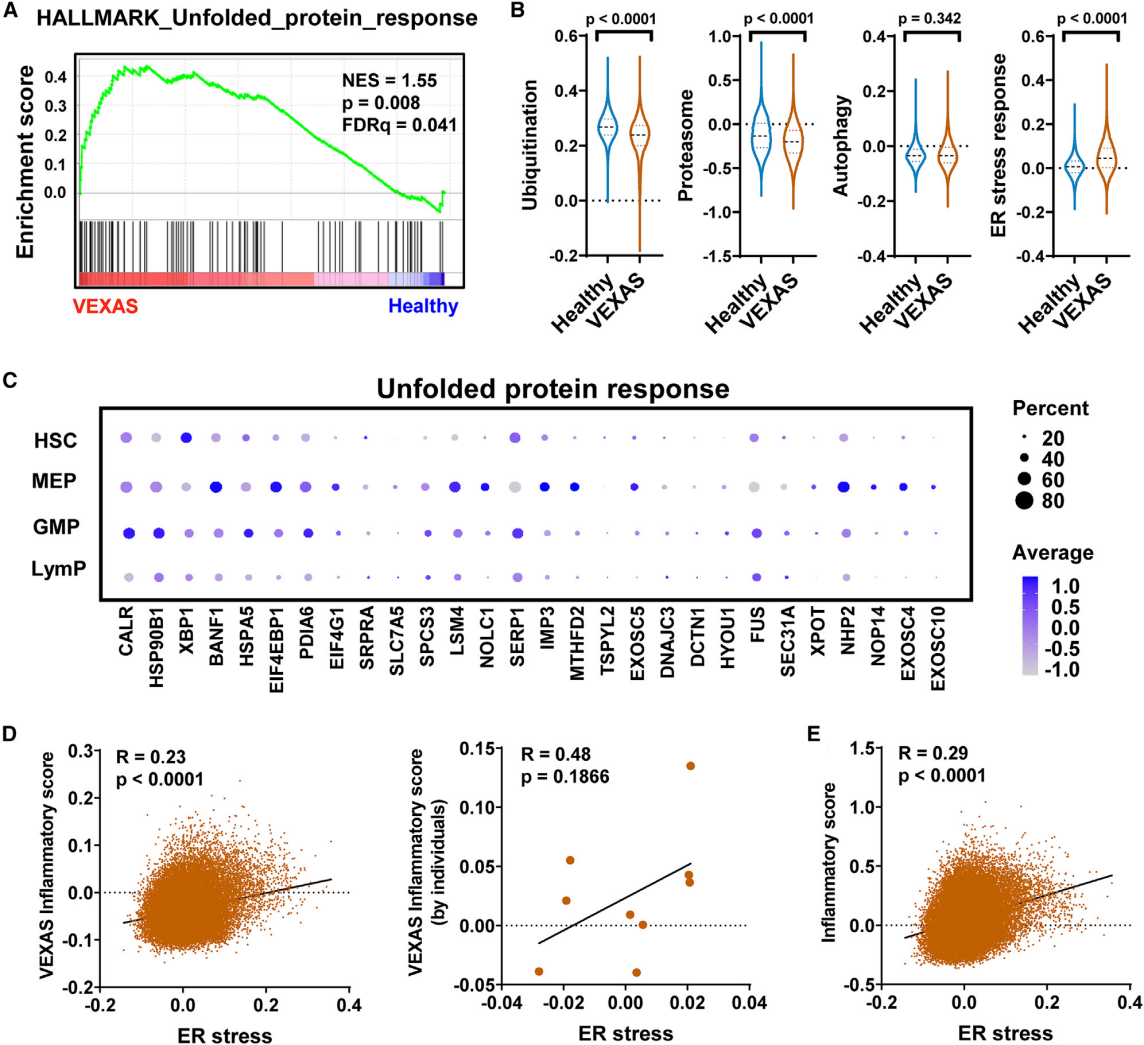
Dysregulated protein degradation and stress response in VEXAS HSPCs (A) A GSEA enrichment plot for a hallmark_unfolded protein response gene set for differentially expressed genes of mt*UBA1* HSPCs compared with wt*UBA1* HSPCs in VEXAS patients. GSEA was based on the Kolmogorov-Smirnov test. (B) Expression levels of pathways (protein ubiquitination, proteasome, autophagy, and response to ER stress) in HSPCs of VEXAS patients (n = 9) and healthy controls (n = 4). p values with the two-sided unpaired t test are shown. (C) Bubble plot showing expression of genes in the unfolded protein response pathway in HSPC subsets in VEXAS patients. Dot sizes correspond to percentages of cells expressing genes, and dot colors correspond to expression levels of genes. (D) Correlation of a VEXAS inflammatory score (calculated based on a gene list of IFN-γ and IFN-α signaling, TNF-α via NF-κB signaling, and the inflammatory response pathways) and ER stress on single-cell levels (left, each dot indicates one cell) and in individual patients (right, each dot indicates one patient). p values and R value estimated using a Pearson correlation test are shown. (E) Correlation of an inflammatory score^49^^,^^50^ and ER stress on single-cell levels. Each dot indicates one cell. p values and R value estimated using a Pearson correlation test are shown.

An unfolded protein response gene set was upregulated only in the CD34^+^ HSPC compartment in VEXAS BMMNCs (Figure 4A). To assess functional changes in protein degradation (the protein ubiquitination/proteasome pathway and the autophagy pathway), we compared the expression of these genes in VEXAS HSPCs with that in healthy HSPCs; there was decreased expression of the protein ubiquitination/proteasome pathway, no significant changes of the autophagy pathway, but a marked increase of ER stress response genes (Figure 4B). Upregulation of unfolded protein response genes (including *CALR*, *HSP90B1*, *XBP1*, *BANF1*, and *HSPA5*) was observed in HSPC subtypes (Figure 4C). With observation of consistent upregulation of the IFN-γ response, TNF-α response, and inflammatory response pathways in BMMNCs, HSPCs, and mt*UBA1* cells in VEXAS, we provisionally defined expression of genes involved in these four pathways as a “VEXAS inflammatory signature” and sought correlation of a VEXAS inflammatory score with ER stress in HSPCs. There was a strong positive correlation of the VEXAS inflammatory score with ER stress at a single-cell level (Figure 4D, left), and the same trend for individual patients (Figure 4D, right). More broadly, there was a positive correlation of a more general inflammatory score^49^^,^^50^ with ER stress in VEXAS HSPCs (Figure 4E). These results suggest a dysregulated protein ubiquitination/proteasome pathway due to *UBA1* mutations, and a lack of the compensatory autophagy pathway for protein degradation leads to an increased unfolded protein stress, which likely contributes to enhanced inflammation in VEXAS HSPCs.

### Biased lineage specification, increased apoptosis in mt*UBA1* LymPs, and progressive loss of lymphocytic cells

**Figure 5:**
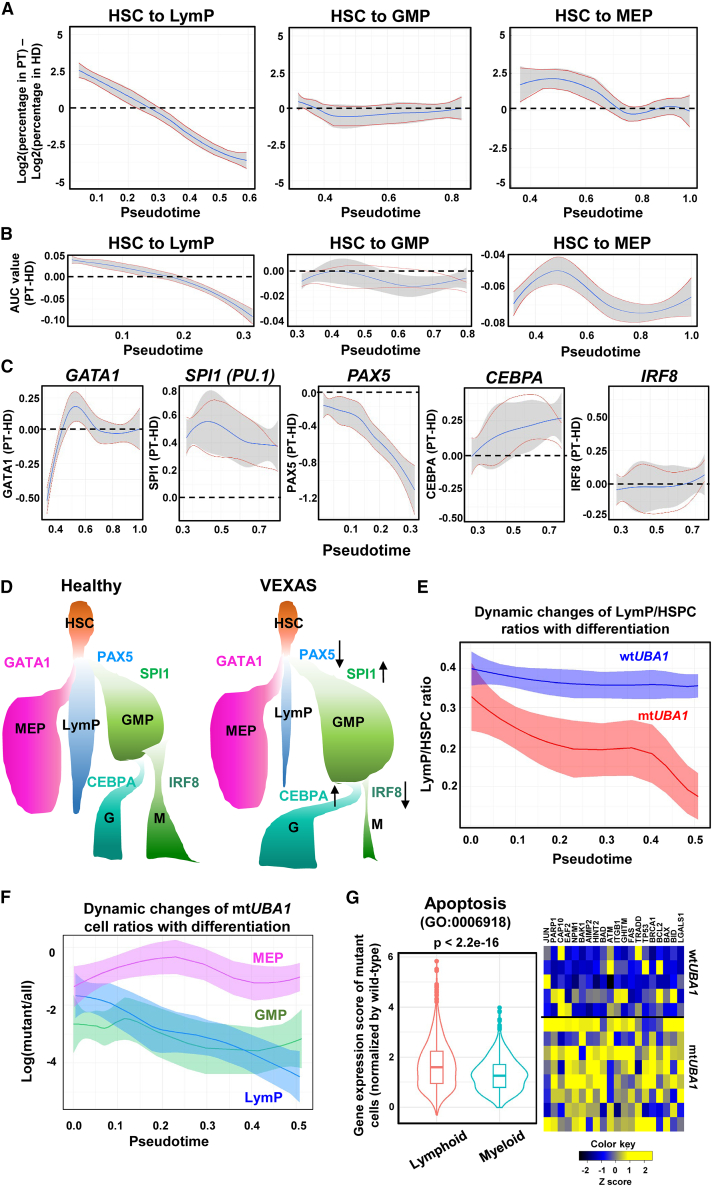
Lineage bias, increased cell apoptosis in mt*UBA1* LymPs, and progressive loss of lymphocytic cells with differentiation (A) Dynamic changes of LymP, GMP, and MEP ratios in VEXAS patients and healthy donors along pseudotime differentiation. x axis: pseudotime ordering from HSCs to LymP, GMP, and MEP, respectively, estimated by Palantir. y axis: Log2(percentages of corresponding cells in VEXAS patients) − log2(percentages of corresponding cells in healthy donors). (B) Dynamic changes of lineage priming of HSCs to LymP, GMP, and MEP, along pseudotime differentiation. x axis: pseudotime ordering from HSCs to lineage-restricted progenitors estimated by Palantir. y axis: Log(lineage signature gene expression in patients/lineage signature gene expression in healthy donors). Lineage signature gene expression represented area under the receiver operating characteristic curve (AUC) values calculated with AUCell. (C) Dynamic changes of expression levels of transcription factors (*GATA1*, *SPI1*, *PAX5*, *CEBPA*, and *IRF8*) along pseudotime differentiation. x axis: pseudotime ordering from HSCs to MEP, GMP, and LymP, respectively, estimated by Palantir. y axis: expression of transcription factors in patients normalized by that in healthy donors. (D) A schematic diagram showing hematopoietic lineage specification and relative quantity of cell types in VEXAS patients and healthy donors. (E) Dynamic changes of wt*UBA1* LymP and mt*UBA1* LymP ratios in HSPCs in VEXAS patients along differentiation. x axis: pseudotime ordering from HSCs to lineage-restricted progenitors estimated by Palantir. y axis: ratios of wt*UBA1* LymP or mt*UBA1* LymP in HSPCs. (F) Dynamic changes of mt*UBA1* cell ratios in LymP, GMP, and MEP in VEXAS patients along differentiation. x axis: pseudotime ordering from HSCs to lineage-restricted progenitors estimated by Palantir. y axis: Log(cell numbers of mutant/all HSPCs). (G) Apoptosis gene expression scores (calculated by the AddModuleScore function in Seurat) of mt*UBA1* cells normalized by wt*UBA1* cells in myeloid and lymphoid BMMNCs were compared. y axis: normalized expression levels of apoptosis genes. A heatmap of apoptosis genes upregulated in *mtUBA1* cells is shown on the right.

To determine whether loss of lymphoid cells occurred early or late in differentiation, we plotted the ratios of patients’ versus healthy donors’ cells along pseudotime differentiation trajectories of different lineages. There was progressive loss of lymphoid cells with differentiation in comparison with normal hematopoiesis, while the numbers of GMP and MEP were stable (Figure 5A). Indeed, a potential of HSC differentiation to lymphocytes progressively decreased; a differentiation potential to myeloid cells remained stable and equivalent to normal hematopoiesis, while a potential to erythroid/megakaryocytes varied but was lower than in normal hematopoiesis (Figure 5B); results were consistent with frequent anemia and lymphocytopenia in VEXAS. We next assessed expression of master transcription factors in hematopoietic lineage specification in VEXAS. At the first lineage specification from stem cells and multipotent progenitors, *GATA1* (to MEP) appeared equivalent to healthy donors, while *SPI1* (encoding PU.1, to GMP) remained higher than in healthy donors, and *PAX5* (to LymP) progressively decreased and was much lower than in normal hematopoiesis (Figure 5C). In the second lineage specification from GMP, *CEBPA* (to G) progressively increased to a level higher than normal, while *IRF8* (to M) appeared normal. Indeed, expression of *PAX5* and *GATA1* were decreased while *SPI1* increased in VEXAS HSCs, and expression of *IRF8* was lower and *CEBPA* higher in VEXAS GMP compared with healthy donors (Figures S4G and S4H). These results indicate biased lineage specification in VEXAS HSPCs toward myeloid and against erythroid and lymphoid differentiation, and relative dominance of neutrophil over monocyte differentiation (Figure 5D). Single-cell results were consistent with clinical and laboratory evidence of myeloid dominance and normal neutrophil number, but monocytopenia in VEXAS.

mt*UBA1* frequency also decreased in lymphoid cells with differentiation (Figure 5E), but was stable or increased in myeloid and erythroid/megakaryocytic lineages (Figure 5F). Upregulation of apoptosis genes occurred in almost all lineages in patients’ HSPCs and BMMNCs (Figures 1C and 2F). To correlate mt*UBA1* and wt*UBA1* cells in myeloid and lymphoid lineages with loss of lymphoid cells and myeloid dominance in VEXAS, we compared ratios of apoptosis gene expression in mutated compared with wild-type cells. mt*UBA1* lymphoid cells had a higher ratio than did mt*UBA1* myeloid cells (Figure 5G), suggesting that mutated lymphoid cells were more susceptible to apoptosis than were mutated myeloid cells. Expression of cell apoptosis genes, including *BCL2*, *JUN*, *CASP10*, *PARP1*, and *ATM*, was higher in mt*UBA1* cells.

### Enhanced cell-cell interactions between activated myeloid cells and HSPCs

**Figure 6:**
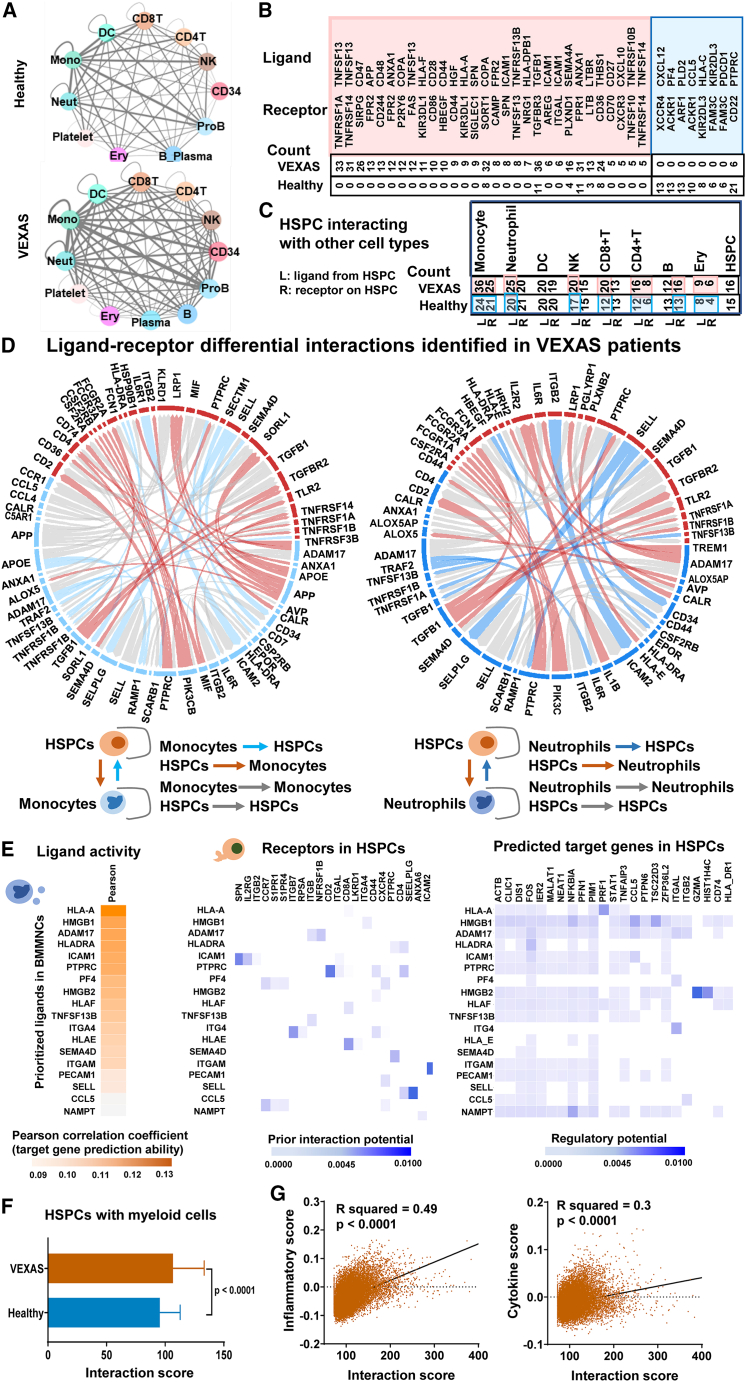
Enhanced cell-cell interactions of activated myeloid cells with HSPCs in VEXAS patients (A) Ligand-receptor pairs among cell types in BMMNCs were estimated by CellPhoneDB.^51^ Color legends for cell types are the same as in Figure 1B. Thickness of lines connecting cell types indicates total number of ligand-receptor pairs between two cell types estimated by CellPhoneDB.^52^ In general, there were more ligand-receptor interactions between cell types in BMMNCs of VEXAS (bottom) than in those of healthy donors (top). CD8T, CD8^+^ T cell; CD4T, CD4^+^ T cell; NK, natural killer cell; CD34, CD34^+^ cell; ProB, pro-B cell; B_Plasma, B cell_Plasma cell; Ery, erythroblast; Neut, neutrophil; Mono, monocyte; DC, dendritic cell. (B) Among the 149 ligand-receptor pairs expressed in VEXAS patients and healthy donors, the number of ligand-receptor pairs presenting in BMMNCs in VEXAS patients and healthy donors were counted. A pink box indicates ligand-receptor pairs most represented in VEXAS; most of them were unique in VEXAS BMMNCs. A blue box indicates ligand-receptor pairs that are less represented in VEXAS than in healthy donors. (C) Summary of ligand-receptor pairs between HSPCs with many immune cell types in VEXAS patients and in healthy donors. Highlights in pink for patients and blue for healthy donors indicate that the number of ligand-receptor pairs is higher in patients than in healthy donors. (D) Potential target genes were identified as differentially expressed in VEXAS patients compared with healthy donors with an adjusted p value < 0.05 and a log fold change > 0.1 or < −0.1. Summary of ligand-receptor differential interactions identified in VEXAS patients by NicheNetr based on differentially expressed genes. Blue segments, molecules expressed by monocytes (left) and neutrophils (right); red segments, molecules expressed by CD34^+^HSPCs; blue arcs, interactions of ligands from monocytes/neutrophils with receptors on CD34^+^HSPCs; red arcs, interactions of ligands from CD34^+^HSPCs with receptors on monocytes/neutrophils; gray arcs, interactions of ligands and receptors in the same cell types. Left and right panels show interactions between monocytes and CD34^+^HSPCs, and between neutrophils and CD34^+^HSPCs, respectively. (E) Cell-cell interactions were defined by NicheNetr.^53^ Ligands expressed by BMMNCs were ranked by likelihood that ligands would affect gene expression changes in CD34^+^HSPCs. Receptors expressed on CD34^+^HSPCs were selected based on their known potentials to interact with prioritized ligands. Finally, target genes were selected based on their differential expression in CD34^+^HSPCs and their potentials to be regulated by ligand-receptor interactions identified between BMMNCs and CD34^+^HSPCs. (F) Interaction scores of patients’ CD34^+^HSPCs with myeloid cells were compared with those in healthy donors. p values with the two-sided unpaired t test are shown. (G) Correlation of interaction scores (CD34^+^HSPCs with myeloid cells in patients) with inflammatory scores (left) and cytokine scores (right) were analyzed. p values and R values with the Pearson correlation test are shown.

We first examined interactions among cell types in BMMNCs. Among 149 ligand-receptor pairs expressed in VEXAS and healthy donors, there were in total 2,488 cell type ligand-receptor pairs among BMMNC cell populations in VEXAS and 2,014 in healthy donors. In general, cell-cell interactions among populations of BM were higher in VEXAS (Figure 6A). The most differentially present ligand-receptor pairs included TNFSF13-TNFRSF1A, TNFSF13-TNFRSF14, CD47-SIRPG, APP-FPR2, TNFSF13-FAS, HLA-F-KIR3DL1, and HLA-A-KIR3DL1. Among them, most were uniquely present in VEXAS and absent in healthy donors (Figure 6B). The most frequent ligand receptors were among immune cells including neutrophils, monocytes, dendritic cells, NK cells, and T cells. HSPCs appeared to interact frequently with diverse immune cells, and interactions of HSPCs with most cell types were higher in VEXAS than in healthy donors (Figure 6C).

NicheNet is a novel algorithm that employs gene expression data to impute ligand-receptor interactions that mediate downstream transcriptional changes by integrating pre-existing knowledge of signaling and regulatory networks.^53^ NicheNet was applied to model interactions between monocytes/neutrophils and CD34^+^ HSPCs, which could potentially induce differential expression of target genes in VEXAS (Figure 6D). For monocyte/neutrophil (ligand)-HSPC (receptor) interactions, top predicted ligands expressed by monocytes/neutrophils were ADAM17, SEMA4D, HLA-DRA, and TNFSF13B, and top receptors expressed by HSPCs were P2RY13, ITGA4, and PLXNB2; IL-6R and IL-1B also were highly expressed in monocytes/neutrophils in interactions with HSPCs. From a map of predicted target genes that were differentially expressed in HSPCs in VEXAS patients to a ligand-receptor activity heatmap (Figure 6E), ligand-receptor interactions between immune cells and HSPCs were consistent with upregulation of genes including *STAT1*, *TNFAIP3*, *ITGAL*, and *GZMA* involved in inflammation in HSPCs. In summary, cell-cell interaction analysis revealed enhanced interactions of myeloid cells with HSPCs and across most cell types in the BM, and involving frequent IFN and TNF interactions with their receptors. Interaction scores of HSPCs with myeloid cells were elevated in VEXAS (Figure 6F). Inflammatory scores and cytokine scores^49^^,^^50^ for each cell were calculated to evaluate inflammation in single progenitor cells: HSPCs with higher interaction scores with myeloid cells had higher inflammatory and cytokine scores (Figure 6G).

### Lymphocyte clonal expansion in VEXAS

**Figure 7:**
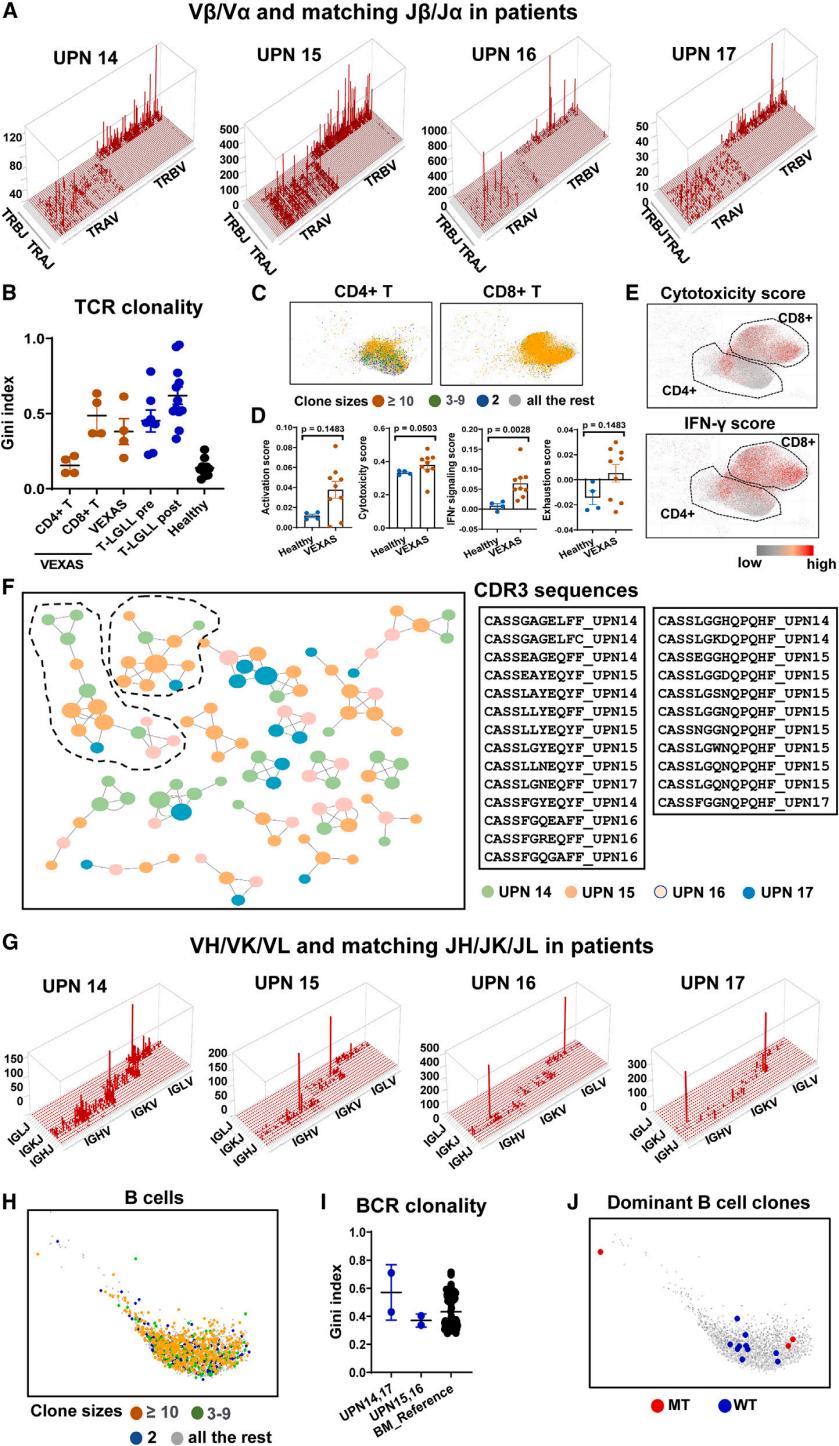
TCR and BCR usage in VEXAS (A) Skyscraper plots showing Vβ/Vα and matching Jβ/Jα in VEXAS patients (UPNs 14–17). (B) Gini indexes of TCR clonality in CD4^+^ T cells, CD8^+^ T cells, and total T cells of BM of VEXAS patients (n = 4), peripheral blood T cells of T-large granular lymphocytic leukemia (T-LGLL) patients pre-aleumtuzmab treatment (n = 13), T-LGLL patients post-aleumtuzumab treatment (n = 12), and healthy donors (n = 7).^56^ (C) Clone size information was projected to the UMAP of CD4^+^ T and CD8^+^ T cells in VEXAS patients. Clones with size 2 (two cells with identical TCR sequences) are in blue color, clones with sizes 3–9 (three to nine cells with identical TCR sequences) in green, and highly expanded clones with sizes ≥ 10 (at least 10 cells with identical TCR sequences) in red, and all other cells in gray. (D) Expression of T cell activation score, cytotoxicity score, IFN-γ signaling score, and exhaustion score were plotted by individual for patients (n = 9) and healthy donors (n = 4). p values with the two-sided unpaired Mann-Whitney test were shown. (E) CD4^+^ and CD8^+^ T cells from VEXAS patients were plotted in UMAP. Top 10% cells expressing the highest cytotoxicity score and IFN-γ signaling score are highlighted in red and all the rest in gray. (F) TCRs identified in VEXAS patients were clustered by GLIPH2, and clusters with at least four TCRs are shown. Colors indicate TCR sequences originated from individual patients. CDR3 sequences for the top two largest TCR clusters are listed, encompassing TCRs from all four patients. (G) Skyscraper plots showing VH/VK/VL and matching JH/JK/JL in VEXAS patients (UPNs 14–17). (H) Clone size information was projected to the UMAP of B cells in VEXAS patients. Clones with size 2 (two cells with identical BCR sequences) are in blue color, clones with sizes 3–9 (three to nine cells with identical BCR sequences) in green, highly expanded clones with sizes ≥ 10 (at least 10 cells with identical BCR sequences) in red, and all other cells in gray. (I) Gini indexes of BCR clonality in UPNs 14–17, and healthy donors in a reference study (n = 71).^57^ Data are presented as mean values ± SEM. p values with two-sided unpaired Mann-Whitney test are shown. (J) B cells from the two largest clones are plotted on UMAP. Clone CAKVYSGEMATMFGFDHSHYYGMDVW (size 449) and clone CARNLLMWFGEFYPW (size 186). B cells with captured *UBA1* mutations are highlighted in red; B cells with captured wild-type *UBA1* transcripts and all the rest are highlighted in gray.

We unexpectedly observed clonal TCR rearrangements in UPNs 14–17, despite patients in our cohort not manifesting clinical evidence of T cell clonal expansion. Skyscraper plots showed TCR Vβ/Vα and matching Jβ/Jα in UPNs 14–17 (Figure 7A). The Gini index measures equality of distribution,^54^^,^^55^ and, for TCR/BCR diversity, the Gini index correlates positively with T/B cell clonality. We calculated Gini indexes of distribution of Vβ sequence clone sizes to quantify TCR clonality in UPNs 14–17, and compared them with published data.^56^ Gini indexes of TCRs in UPNs 14–17 were higher than in healthy donors (Figure 7B), indicating restricted TCR usage in UPNs 14–17, but there was little sharing of TCR clones among these four VEXAS patients, nor with healthy donors or T-large granular lymphocytic leukemia patients (Figure S5).^56^ T cell clonal expansion was mainly among CD8^+^ T cells compared with CD4^+^ T cells (Figures 7B and 7C). Cytotoxicity and IFN-γ response in VEXAS T cells were increased (Figure 7D) and predominantly in CD8^+^ T cells (Figure 7E). Using GLIPH2, we sought similar TCRs among these four patients, and the top TCR clusters were composed of TCR sequences from the patients, suggestive of common antigens in VEXAS, but this analysis was limited by a the small sample size.

We examined BCR rearrangement in UPNs 14–17 (Figure 7G): UPNs15 and 16 had plasma cell myeloma and a small CD5^+^ B cell clone, respectively. B cell clonal expansion was observed in VEXAS (Figure 7H) but to a similar level as in healthy donors. Gini indexes of BCRs in UPNs 14 and 17 were not different from those in healthy donors^57^ and Gini indexes of BCRs in UPNs 15 and 16 were not higher than those in UPNs 14 and 17, despite clinical evidence of plasma cell or B cell dyscrasia (Figure 7I). We examined BCR usage in UPNs 14–17: there was no overlap among UPNs 14–17 nor with BCRs of healthy individuals^57^ (Figure S6). When we linked *UBA1* mutation information with BCR sequences in the same cell, BCR expression was detected in hundreds of cells in UPNs 14 and 17, but expressed wt*UBA1* and mt*UBA1* transcripts were present in only a few cells, due to the low mutation frequency in lymphoid cells and technical dropout. mt*UBA1* and wt*UBA1* cells were present in the same BCR clones (Figures 7J and S7).

## Discussion

VEXAS has been described only recently, but its unusual etiology was clear in the original report^1^: an acquired mutation in hematopoietic stem cells, found only in men due to the X chromosome location of the *UBA1* gene, resulting in severe, multiorgan autoinflammation that manifested in a range of familiar rheumatologic diagnoses. Zebrafish knockouts of the homologous gene suggested that inflammation was a direct consequence of the mutations,^1^ but other attempts to model VEXAS in animals and *in vitro* have proven difficult in practice, perhaps due to the fundamental cellular role of the ubiquitylation pathway disrupted by an altered *UBA1* gene product. Nevertheless, pathophysiologic mechanisms have been unclear: how gene mutations lead to inflammation, which cell types are important in effecting tissue damages, and the routes to both lymphocyte depletion and malignant plasma cell proliferation. Other basic questions are the discordance between mt*UBA1* cell clonal dominance in patients and deficient hematopoietic cell proliferation *in vitro*, and how altered protein degradation relates to the initiation of profound upregulation of multiple innate immune pathways and globally elevated cytokine production.

Here, we utilize single-cell methodologies to characterize transcriptomes and expressed mutations of BM precursors and HSPCs from VEXAS patients, in a comprehensive and unbiased approach to characterize this poorly understood disease. At the high resolution of scRNA-seq, activation of multiple different inflammatory pathways were striking in primitive stem and progenitor cells. The protein ubiquitination and proteasome pathway were dysregulated, with no apparent compensation by autophagy to allow protein degradation, likely leading to elevated stress response in VEXAS, as suggested by our data and previous studies.^58^ Mutations in *UBA1* not only resulted in marked activation of the innate immune pathways (implying a cell-autonomous mechanism resulting from *UBA1* disruption), but they altered cell cycling, potentially providing a mechanism for clonal dominance. Lineage specification of HSCs, governed by several master transcription factors, was skewed toward myeloid, and especially granulocytic differentiation and against lymphoid differentiation in HSCs in VEXAS, and there was progressive loss of lymphocytes with differentiation, accompanied by increased apoptosis restricted to the lymphoid trajectory. Myeloid lineage cells had the most inflammatory activation, but lymphoid cells, predominantly wt*UBA1*, also had elevated expression to a lesser extent of the same genes. Increased cell-cell interactions between myeloid cells and HSPCs and among major cell types in BM in VEXAS revealed enhanced “crosstalk” in this inflammatory environment, particularly for the IFN and TNF-α pathways. Taken together, our results begin to define the characteristics of inflammation, lineage disequilibrium, and genotype association at single-cell molecular resolution for VEXAS syndrome.

One general hypothesis to explain clonal hematopoiesis is that cells with pre-existing somatic mutations are selected due to fitness in their microenvironments, as for example, the familiar loss of HLA gene expression as an escape from immune destruction.^59^^,^^60^^,^^61^ An alternative, not mutually exclusive mechanism, is that an acquired mutation contributes to or even drives chronic inflammation, as has been proposed for proinflammatory *TET2* mutations in CHIP, with consequent proinflammatory tissue-resident macrophages and accelerated atherosclerotic events.^62^^,^^63^^,^^64^ Conversely, chronic infection and inflammation appear to secondarily favor expansion of mt*DNMT3A* somatic clones, perhaps due to their bias toward self-renewal over terminal differentiation in a stressed or regenerating environment, as has been inferred from murine models.^65^^,^^66^ *DNMT3A* mutations also alter immune phenotypes^67^; in a recently published single-cell multi-omics study, upregulation of a few genes involved in proinflammatory signaling is observed in mt*DNMT3A* HSPCs.^33^ Our data strongly favor *UBA1* mutations in HSPCs as immediate drivers of both myeloid lineage dominance and the origin of inflammation in VEXAS syndrome. Nevertheless, with apoptosis genes upregulated and cell cycling genes downregulated in BMMNCs and HSPCs in VEXAS, and functionally, both BMMNCs and CD34^+^ HSPCs from patients formed fewer colonies than did cells from healthy individuals (Figure S7B), myeloid progenitors and HSPCs in VEXAS likely exhibited defective proliferation and differentiation, reflecting the ineffective hematopoiesis typical of MDS.

In addition, the hyperinflammatory microenvironment in VEXAS syndrome likely does positively select other somatically mutated clones: *DNMT3A* and *TET2* clones are frequent in VEXAS^18^^,^^19^^,^^20^^,^^21^ as they are in other inflammatory clinical conditions.^68^ We also characterized the transcriptome of mt*DNMT3A* cells in the setting of VEXAS. Upregulation of the inflammation and immune response pathways in mt*DNMT3A* cells in our VEXAS cases indicated a proinflammatory phenotype associated with this epigenetic genotype, in accord with previous reports^33^^,^^67^; cell cycling of mt*DNMT3A* cells appeared to be decreased compared with wt*DNMT3A* cells. Whether mt*UBA1* cells might also have a selective advantage in an inflammatory environment—possibly creating a deleterious autocrine/paracrine loop—has been unclear, but we noted marked activation of multiple inflammatory pathways early in hematopoietic ontogeny, in contrast to CHIP-mutated inflammatory terminal cells. In addition, the allele frequency of mt*UBA1* clones did not correlate with clinical severity of inflammation in patients. Our analysis demonstrated a positive correlation between interaction of mt*UBA1* HSPCs with myeloid cells and inflammation: with enhanced cell-cell interactions in VEXAS, malicious feedback may exaggerate inflammation in the disease. The marked involvement of the IFN-γ and TNF-α pathways, and cell-cell interactions mediated by IFNs, TNF-α, IL-1β, and IL-6 suggest that IFN and TNF blockers or disruption of cell-cell interactions are potential targets of therapies in VEXAS. In addition, diagnostic distinctions may be aided by application of single-cell genomic results: both marked activation of several critical inflammatory pathways and activation in primitive HSPCs may be useful in distinguishing among VEXAS syndrome and other hematologic diseases with overlapping clinical features.

Plasma cell dyscrasias are frequent in VEXAS,^1^^,^^3^^,^^4^ despite absence of *UBA1* mutations in lymphocytes. Diversity of BCR repertoires in two of our patients with clinical evidence of B/plasma cell clonal expansion was similar to that in other VEXAS patients and both were similar to BCR clonality reported in healthy individuals,^57^ indicating that BCR usage was preserved in disease, but with reduced B cell numbers and distinct BCR repertoires across individuals. Linking *UBA1* mutations with BCR sequences in single cells showed coexistence of mt*UBA1*and wt*UBA1* cells within the same BCR clone, implying antigenic drivers rather than the *UBA1* mutation itself were driving B cell expansion. Unexpected was reduced TCR diversity detected by scRNA-seq in four patients, as to date there are no reports of TCR clonal expansion in VEXAS. Furthermore, T cells, especially clonally expanded CD8^+^ T cells in VEXAS marrow had increased cytotoxicity and IFN-γ signaling, and one inference is T cells targeting unknown antigens could be actively involved in disease pathogenesis. A higher percentage of activated CD8^+^ T cells was observed in relapsing polychondritis patients with VEXAS compared with polychondritis patients without VEXAS and healthy controls.^5^ Oligoclonal B and T cells (reduced in number and mainly unmutated) may be secondary to immune cell recognition of novel, aberrant, or overexpressed antigens, and specific presentation of immunogenic epitopes due to abnormal protein degradation.

In conclusion, our study of single-BM hematopoietic cells facilitates understanding of the pathophysiology of the newly defined disease VEXAS. Specifically, simultaneous genotyping and phenotyping, and direct comparison of mutated and wild-type human HSPCs provide fundamental insights in the direct and indirect roles of *UBA*1 mutations in VEXAS pathophysiology. Our results expand our knowledge of distinct transcriptome signatures and crosstalk among BM hematopoietic cells, *UBA1* and *DNMT3A* mutations, and TCR/BCR repertoires in VEXAS.

### Limitations of the study

Our study has limitations. First, the sample size of the explorative cohort was relatively small (due to the high cost of experiments) and potentially biased by patients with rheumatologic manifestations (due to referral patterns at our center). Nevertheless, observations from scRNA-seq were reproducible in an independent validation cohort with traditional immunological methods. However, attempts at confirmation by flow cytometry and ELISpot with HSPCs were not successful due to low cell numbers. Second, detection of *UBA1* and *DNMT3A* mutations with scRNA-seq data was limited by low transcript abundance, allelic and technical dropout, and incomplete transcript coverage inherent to the platform.^48^ Indeed, very few experimental protocols have been published that allow reliable simultaneous RNA and DNA sequencing of single cells.^31^^,^^32^^,^^33^ Due to the prospective nature of the study and the limitation of 3′- or 5′-biased sequencing reagents, completely reliable simultaneous detection of *UBA1* and *DNMT3A* mutations in single cells was not achieved. Technologies under development that enable simultaneous sequencing of both RNA and DNA of single cells at high throughput^31^^,^^32^^,^^33^ may help to define the clonal architecture of *UBA1* and other somatic mutations frequent in VEXAS, and allow correlation of transcriptome signatures in multiply mutated single cells. Third, although we performed knockdown *in vitro* experiments with human cell lines, validation in primary human cells and animal models is desirable, if not yet achievable.

## STAR★Methods

### Key resources table

**Table:** 

REAGENT or RESOURCE	SOURCE	IDENTIFIER
**Antibodies**		

Anti-human lineage cocktail (Clones UCHT1, HCD14, 3G8, HIB19, 2H7, and HCD56) Pacific blue	BioLegend	Cat# 348805; RRID: AB_2889063
Mouse anti-human CD34 (Clone 581) PE	BD Biosciences	Cat# 555822; RRID: AB_396151
Mouse anti-human CD38 (Clone HIT2) APC	BD Biosciences	Cat# 555462, RRID: AB_398599
Anti-human CD90 (Clone 5E10) FITC	BioLegend	Cat# 328108, RRID: AB_893429
Anti-human CD10 (Clone HI10A) BV605	BD Biosciences	Cat# 562978, RRID: AB_2737929
Anti-human CD135 (Clone BV10A4H2) PE/Cyanine7	BioLegend	Cat# 313314, RRID: AB_2565478
Anti-human CD45RA (Clone HI100) BV510	BioLegend	Cat# 304142, RRID: AB_2561947
Human IFN-γ/TNF-α Double-Color Enzymatic ELISPOT Assay kit	ImmunoSpot	Cat# SKU:hIFNgTNFa-2M

**Biological samples**		

10% fetal bovine serum	Sigma-Aldrich	Cat# 12306C
Healthy bone marrow sample	National Institutes of Health	Healthy bone marrow sample
VEXAS patients’ bone marrow sample	National Institutes of Health	VEXAS patients’ bone marrow sample

**Chemicals, peptides, and recombinant proteins**		

LSM Lymphocyte Separation Medium	MP Biomedicals	Cat# 50494X
Phosphate buffered saline	Lonza	Cat# 17-516Q
ACK lysing buffer	Quality Biological	Cat# 118-156-101
IMDM	Thermo Fisher Scientific	Cat# 12440053
MethoCult™ H4434 Classic semisolid methylcellulose medium	STEMCELL Technologies	Cat# 04444
MethoCult™ H4435 Enriched semisolid methylcellulose medium	STEMCELL Technologies	Cat# 04445
RPMI 1640	Thermo Fisher Scientific	Cat# 11875093
Polybrene	Sigma-Aldrich	Cat# TR-1003

**Critical commercial assays**		

10x Genomics System using the Chromium Single Cell 3′ Reagent Kit v2	10x Genomics	Cat# 120237
10x Genomics Single Cell Immune Profiling Solution v 1.1	10x Genomics	Cat# 1000165

**Deposited data**		

Raw and analyzed data	This paper	GEO: GSE196052
Published scRNA-seq data of HSPCs in MDS patients	Compare inflammatory signaling scores with published data Ganan-Gomez et al. 2022^37^	GEO: GSE137429
Published scRNA-seq data of HSPCs in CMML patients	Compare inflammatory signaling scores with published data Wiseman et al. 2020^38^	Array Express: E-MTAB-8884
Published scRNA-seq data of HSPCs in CML patients	Compare inflammatory signaling scores with published data Giustacchini et al. 2017^39^	GEO: GSE76312
A public database of B cell receptor sequences	Compare BCR usage with published data DeWitt et al. 2016^57^	http://adaptivebiotech.com/pub/robins-bcell-2016
Published T cell receptor sequences in T-LGLL patients and healthy donors	Compare TCR usage with published data Gao et al. 2022^56^	GEO: GSE168859

**Experimental models: Cell lines**		

293T cells	ATCC	CRL-3216
U937 cells	ATCC	CRL-3253
THP-1 cells	ATCC	TIB-202
Raji cells	ATCC	CCL-86
Jurkat cells	ATCC	TIB-152

**Oligonucleotides**		

pLKO1-Puro Mission shRNA constructs	Sigma-Aldrich	KD1 TRCN0000004003
pLKO1-Puro Mission shRNA constructs	Sigma-Aldrich	KD2 TRCN0000277770,
pLKO1-Puro Mission shRNA constructs	Sigma-Aldrich	KD3 TRCN0000277769
pLKO1-Puro Mission shRNA constructs	Sigma-Aldrich	KD4 TRCN0000004004

**Recombinant DNA**		

Lentiviral construct pLKO-1 puro plasmid	Addgene	Cat# 8453
Packaging plasmid pCMV-VSV-G	Addgene	Cat# 8454
Packaging plasmid pRSV-Rev	Addgene	Cat# 12253
Packaging plasmid pHM-Tat1b	Addgene	Cat# 164442

**Software and algorithms**		

cellranger count version 3.0.1	10x Genomics	https://support.10xgenomics.com/singlecell-gene expression/software/downloads/3.0/
cellranger vdj version 3.0.1	10x Genomics	https://support.10xgenomics.com/singlecell-gene-expression/software/downloads/3.1/
Seurat version 2.3.4	Stuart et al., 2019^70^	https://cran.r-project.org/web/packages/Seurat/index.html
R version 3.5.0	R Core Team, 2021^70^	https://www.r-project.org/
cb_sniffer version 1.0	Pettti et al., 2019^48^	https://github.com/sridnona/cb_sniffer
CellPhoneDB version 3.1.0	Garcia-Alonso L et al. 2022^71^	https://www.cellphonedb.org/
NicheNet version 1.0.0	Browaeys et al., 2020^53^	https://github.com/saeyslab/nichenetr
fGSEA version 1.16.0	Korotkevich et al., 2019^72^	http://bioconductor.org/packages/release/bioc/ html/fgsea.html
Palantir version 1.0.0	Setty et al.2019^73^	https://github.com/dpeerlab/Palantir
topGO version 2.34.0	Alex, A., and Rahnenfuthrer, J. 2019^74^	https://bioconductor.org/packages/release/bioc/html/topGO.html
AUCell version 1.4.1	Aibar et al., 2017^75^	https://bioconductor.org/packages/release/bioc/html/AUCell.html
tCR version 2.3.2	Github repository	https://imminfo.github.io/tcr/
Genomatix Generanker	Werner et al., 2013^76^	http://www.genomatix.de
STRING version 9	Szklarczyk et al., 2015^77^	https://string-db.org/
GraphPad Prism 9.02	GraphPad software	https://www.graphpad.com/scientificsoftware/prism/
FlowJo v7.6.4	Tree Star	https://www.flowjo.com
Analysis scripts	This paper	https://github.com/shouguog/UBA1

**Other**		

Analysis and visualization of the scRNA-seq datasets in this study can be performed at the interactive website	This paper	https://shouguog.shinyapps.io/vexas_cd34_bm/

### Resource availability

#### Lead contact

Further information and request for resources and reagents should be directed to and will be fulfilled by the lead contact Zhijie Wu (zhijie.wu@nih.gov).

#### Materials availability

This study did not generate new unique reagents.

### Experimental model and study participant details

#### Human samples

Bone marrow (BM) samples were obtained from VEXAS patients after written informed consent under protocol (www.clinicaltrials.gov NCT00001373) approved by the Institutional Review Board of the National Human Genome Research Institute, in accordance with the Declaration of Helsinki. Healthy donors were enrolled as controls under protocol NCT00001620 in National Heart, Lung, and Blood Institute. Four healthy donors (male, 57/61/62/68 years old) were age- and gender-matched with patients in explorative cohort for scRNA-seq analyses.

### Method details

#### Bone marrow processing and cell sorting

BM mononuclear cells (BMMNCs) were isolated, followed by flow cytometric sorting to enrich lineage CD34^+^ hematopoietic stem and progenitor cells (HSPCs); both BMMNCs and HSPCs were used for single-cell RNA sequencing (scRNA-seq). Fresh BM samples were processed within 16 h, followed by either direct analyses (flow cytometry and colony forming assay for all individuals; scRNA-seq for UPNs 1, 6, 10, and 11) or cryopreserved until use (UPNs 14–17) to enrich lineage-CD34^+^ HSPCs and for scRNA-seq. Another 11 patients and 8 healthy donors were enrolled in a validation cohort, with cryopreserved BMMNC samples primarily for immunophenotyping and ELISpot assays.

BM specimens were obtained from patients and healthy donors and kept in heparin tubes, and processed within 16 h after collection. BMMNCs from each person were isolated by density centrifugation using LSM Lymphocyte Separation Medium (Cat# 50494X, MP Biomedicals). Briefly, BM was diluted 2-fold using phosphate buffered saline (PBS) (Cat# 17-516Q, Lonza), layered on top of 1 volume LSM Lymphocyte Separation Medium in a 50-mL Falcon tube, and spun down at 1,140*g* for 25 min at room temperature with brake off. A BMMNC layer was isolated and washed with PBS after red blood cell lysing with ACK lysing buffer (Cat# 118-156-101, Quality Biological). BMMNCs were resuspended in the IMDM (Cat# 12440053, Thermo Fisher Scientific) + 2% fetal bovine serum (Cat# 12306C, Sigma-Aldrich) before fluorescence-activated cell sorting (FACS) to enrich lineage^−^CD34^+^ hematopoietic stem and progenitor cells (HSPCs). BMMNCs were stained with monoclonal antibodies (Abs) for 30 min on ice: anti-human lineage cocktail (CD3, CD14, CD16, CD19, CD20, and CD56; clones UCHT1, HCD14, 3G8, HIB19, 2H7, and HCD56, respectively, Cat# 348805, BioLegend) in Pacific Blue; anti-CD34 Ab (clone 581, Cat# 555822, BD Biosciences) in PE, and anti-CD38Ab (clone HIT2, Cat# 555462, BD Biosciences) in APC. Cells were sorted using the FACSAria Fusion Flow Cytometer (BD Biosciences). Aliquots of BMMNCs were subjected to multi-color flow cytometry to profile HSP subpopulations. BMMNCs and purified lineage^−^CD34^+^ cells were subjected to colony forming assay and scRNA-seq analysis.

#### Human primary cell culture

BMMNC isolated from fresh BM were used for flow cytometry, cell sorting, and scRNA-seq were proceeded without cell culture. Cell culture conditions for primary BM cells used for colony forming assay and ELISpot assay were described in “Colony forming assay” and “ELISpot assay to check IFN-γ and TNF-α secreted by human BMMNCs” sections below, respectively, with different cell culturing conditions per experiments. In brief, for colony forming assay, isolated BMMNCs and sorted CD34^+^ cells were cultured in semisolid methylcellulose medium at 37°C with 5% CO_2_ for 14 days. For ELISpot assay, BMMNCs were cultured in CTL-Test Medium in 96-well plates and incubated in a 37°C humidified incubator, 5–9% CO2 for 20 h.

#### Colony forming assay

BMMNCs from individuals were mixed in semisolid methylcellulose medium (MethoCult H4434 Classic, Cat# 04444, STEMCELL Technologies) containing interleukin (IL)-3, stem cell factor (SCF), erythropoietin (EPO), and granulocyte-macrophage colony-stimulating factor (GM-CSF) at 2 x 10^4^ cells/plate. Sorted CD34^+^ cells from individuals were mixed in semisolid methylcellulose medium (MethoCult H4435 Enriched, Cat# 04445, STEMCELL Technologies) containing IL-3, IL-6, SCF, EPO, granulocyte colony-stimulating factor (G-CSF), and GM-CSF at 500 cells/plate. Cells were cultured at 37°C with 5% CO_2_. Colonies were counted at day 14.

#### Flow cytometry profiling of HSPCs

Flow cytometric sorting of lineage^−^CD34^+^ HSPCs following isolation of BMMNCs. BMMNCs were stained with antibody mixtures on ice for 30 min in RPMI 1640 (Cat# 11875093, Thermo Fisher Scientific). Samples were subsequently acquired using the BD LSR Fortessa cytometer (BD Biosciences), and post-acquisition analysis was performed using Flowjo software (v.7.6.4; Flowjo LLC, BD Biosciences). Antibodies used for flow cytometry analyses were: anti-human lineage cocktail (CD3, CD14, CD16, CD19, CD20, and CD56; clones UCHT1, HCD14, 3G8, HIB19, 2H7, and HCD56, respectively, Cat# 348805, BioLegend) in Pacific Blue; anti-human CD34 in PE (clone 581, Cat# 550761, BD Biosciences), anti-human CD38 in APC (clone HIT2, Cat# 555462, BD Biosciences), anti-CD90 in FITC (clone 5E10, Cat# 328108, BioLegend), anti-human CD10 in BV605 (clone HI10A, Cat# 562978, BD Biosciences), anti-human CD135 in PE/Cy7 (clone BV10A4H2, Cat# 313314, BioLegend), and anti-human CD45RA in BV510 (clone HI100, Cat# 304142, BioLegend).

#### ELISpot assay to check IFN-γ and TNF-α secreted by human BMMNCs

IFN-γ and TNF-α secretion from BMMNCs of VEXAS patients and healthy donors were measured using the Human IFN-γ/TNF-α Double-Color Enzymatic ELISPOT Assay kit (Cat# SKU:hIFNgTNFa-2M, ImmunoSpot) in two separate experiments in triplicate (4 patients versus 3 healthy donors for a 1^st^ batch, and 5 patients versus 2 healthy donors for a 2^nd^ batch), according to the manufacturer’s protocol. In brief, pre-coated 96-well plates were activated with Human IFN-γ/TNF-α Capture Solution and 70% ethanol on Day 0, and incubated at 4°C overnight. On Day 1, BMMNCs were suspended in CTL-Test Medium and seeded in 96-well plates at a density of 90,000 cells/well (1^st^ batch) or 400,000 cells/well (2^nd^ batch), and incubated in a 37°C humidified incubator, 5–9% CO2 for 20 h. On Day 2, 96-well plates were washed and incubated sequentially with Anti-human IFN-γ/TNF-α Detection Solution, Tertiary Solution, and Blue and Red Developer Solutions. 96-well plates were then air-dried and face down on paper towels on a bench top for more than 24 h before scanning and counting with the CTL ImmunoSpot Analyzers and ImmunoSpot Software.

#### Human leukemic cell lines culture

Human leukemic cell lines U937, THP-1, Raji, and Jurkat were purchased from the American Type Culture Collection (ATCC). U937, THP-1, Raji and Jurkat cell lines were maintained in RPMI-1640 (Cat# 11875093, Thermo Fisher Scientific) in 10% heat-inactivated fetal bovine serum (Sigma-Aldrich), 1% L-glutamine, 100 units/ml penicillin and 100 μg/mL streptomycin (Thermo Fisher) at 37°C with 5–9% CO2.

#### Knockdown of *UBA1* in human leukemic cell lines

pLKO1-Puro Mission shRNA constructs (Sigma-Aldrich) targeting *UBA1* included KD1 TRCN0000004003, KD2 TRCN0000277770, KD3 TRCN0000277769, and KD4 TRCN0000004004 along with control scramble shRNA were used for producing shRNA knockdown. Lentiviruses were produced in 293T cells by co-transfection of the lentiviral construct pLKO-1 puro plasmid (Cat# 8453, Addgene) with packaging plasmids (pCMV-VSV-G, Cat# 8454; pRSV-Rev, Cat# 12253; pHM-Tat1b, Cat# 164442, Addgene) for 48 to 72 h. Infection was carried out with 2 x 10^6^ of U937, THP-1, Raji, or Jurkat cells (ATCC) in a 6-well plate with lentiviruses in the presence of Polybrene (6 μg/mL; Cat# TR-1003, Sigma-Aldrich). For infection of lentiviruses carrying ectopic expression vectors, cells were centrifuged at 1,000 *g* at 30°C for 90 min. After 4 to 6 days of selection with 0.5–1 μg/mL of puromycin for pLKO1-puro-shRNA constructs, cells were analyzed by immunoblotting with UBA1 (Cat# 4891, Cell Signaling Technology) and actin (Cat# 3700, Cell Signaling Technology) antibodies using the protocol previously described (Beck et al., 2020). RNA was extracted from pooled antibiotic resistant clones for each respective shRNA, after confirmation of knockdown by immunoblotting.

#### Cell preparation, whole transcriptome amplification (WTA), cDNA library preparation, and sequencing

scRNA-seq analysis for patients (UPNs 6, 11, 1, 10, and 13) and healthy donors was performed with the 10x Genomics System using the Chromium Single Cell 3′ Reagent Kit v2 (Cat# 120237), according to the manufacturer’s protocol (www.10xgenomics.com).^78^ scRNA-seq coupled with single-cell T cell receptor/B cell receptor sequencing (scTCR/BCR-seq) analysis for UPNs 14–17 was performed with the 10x Genomics System using the 10x Genomics Single Cell Immune Profiling Solution v 1.1 (Chromium Single Cell 5′ Reagent Kit v1.1, Cat# 1000165, 10x Genomics), following the manufacturer’s protocol (www.10xgenomics.com).^78^ Briefly, BMMNCs and FACS-sorted BM lineage^−^CD34^+^ cells were washed with 1X PBS with 0.04% (w/v) bovine serum albumin. Cell concentration and viability were determined using the Countess II Automatic Cell Counter and the trypan blue staining method. Cell loading and capturing were done on the Chromium Controller (10x Genomics). Following reverse transcription and cell barcoding in droplets, emulsions were broken, and cDNA was purified using Dynabeads MyOne SILANE, followed by PCR amplification. Amplified cDNA was then used for both 3′ and 5′ gene expression library construction and TCR/BCR enrichment. For gene expression library construction, the amplified cDNA was fragmented, end-repaired, and double-sided size-selected with SPRIselect beads. For TCR/BCR library construction, TCR/BCR transcripts were enriched from amplified cDNA by PCR. Subsequently, the enriched PCR product was fragmented, end-repaired, and size-selected with SPRIselect beads. The scRNA libraries were pooled together and sequenced on the Illumina NovaSeq system using read lengths of 26-bp read 1, 8 bp i7 index, 98-bp read 2. The single-cell TCR/BCR libraries were sequenced on the Illumina NovaSeq system using read lengths of 150-bp read 1, 8 bp i7 index, 150-bp read 2. Sequencing metrics were summarized in Table S5.

#### scRNA-seq data analysis

##### Preprocessing of scRNA-seq and scTCR/BCR-seq data

Alignment, barcode assignment, and Unique Molecular Identifier (UMI) counting were performed using the cellranger pipeline (http://software.10xgenomics.com/single-cell/overview/welcome).^78^.

After single-cell libraries were sequenced using the Illumina system, cellranger pipeline (support.10xgenomics.com/single-cell-gene-expression/software/pipelines/latest/what-is-cell-ranger" title="https://support.10xgenomics.com/single-cell-gene-expression/software/pipelines/latest/what-is-cell-ranger">https://support.10xgenomics.com/single-cell-gene-expression/software/pipelines/latest/what-is-cell-ranger) was used to process scRNA-seq raw data in order to align reads to the genome, and to generate gene–cell expression matrices. Specifically, sequencing reads were aligned to the hg19 reference genome by STAR with annotation of ENSEMBL. Uniquely aligned reads were used to quantify gene expression levels for all ENSEMBL genes with UMIs. We filtered and removed low-quality cells from further analysis if the number of genes detected was fewer than 500 (low quality, potential fragments) or more than 3,000 (potential doublets). We also excluded those cells with a high percentage of mitochondrial gene reads (>10%),^79^ and remaining single cells were subjected to subsequent data analyses. Sequencing metrics and detailed information are provided in Table S1.

TCR reads were aligned to the GRCh38 reference genome and consensus TCR annotation was performed using the cellranger vdj program (10x Genomics, version 3.0.1). TCR libraries were sequenced at depth of over 2,000 reads/cell, with a final 33418 mean read pairs/cell. On average, 27,053 reads mapped to either the TRA or TRB loci in each cell. TCR annotation was performed using the 10x cellranger vdj pipeline as described at support.10xgenomics.com/single-cell-vdj/software/pipelines/latest/using/vdj" title="https://support.10xgenomics.com/single-cell-vdj/software/pipelines/latest/using/vdj">https://support.10xgenomics.com/single-cell-vdj/software/pipelines/latest/using/vdj. Barcodes with a higher number of UMI counts than those of simulated background were considered as cell barcodes. V(D)J read filtering and assembly were implemented as a previous study.^80^ cellranger firstly trimmed known adaptor and primer sequences from the 5′ and 3′ ends of reads, and then filtered away reads lacking at least one 15-bp exact match against at least one reference segment (TCR, TRA, and TRB gene annotations in Ensembl version 87). Next, cellranger performed *de novo* assembly for each barcode by building a De Bruijn graph of reads independently. The assembler output contig sequences which were assigned at least one UMI. Finally, each assembled contig was aligned against all of the germline segment reference sequences of the V, D, J, C, and 5′ UTR regions. cellranger searched a CDR3 motif (Cys-to-FGXG/WGXG) in a frame defined by a start codon in the L + V region or all 6 frames when the L + V region was absent. A contig was kept and considered as productive if: 1) it fully spanned the V and J segments; 2) there was a start codon in the V region; 3) it contained a CDR3 region in-frame with a V start codon; 4) there were no stop codons in the V-J spanning region. Most cell barcodes contained two matching productive contigs, comprising either a TCRA or a TCRB though it was of biological possibility that fewer productive contigs (low sensitivity) or >2 productive contigs (some cells do contain more than one TCRB or TCRA chain) were associated with one cell barcode.^81^ Similarly, BCR reads were also processed using the cellranger vdj program, with the IMGT database of GRCh38 genome as reference. Only productive contigs of BCR were kept for analysis.

Downstream analyses were performed using the R software^82^ package in Seurat (Stuart et al., 2019; http://satijalab.org/seurat/, v2.3.4)^70^ on BMMNCs and lineage^−^CD34^+^ cells separately (Satija et al., 2015).^83^ Raw reads in each cell were first scaled by a library size to 10,000, and then log-transformed. To improve downstream dimensionality reduction and clustering, regressionOut in the Seurat package^84^ was used to remove unwanted sources of variation based on the number of UMIs and percentages of mitochondrial reads. Highly variable genes (∼1,600 for BM cells and ∼1,900 for CD34^+^ cells, identified with y.cutoff = 0.5) were used for Principal Component Analysis (PCA) of high-dimensional data. Top 30 principal components were selected for unsupervised clustering of cells with a graph-based clustering approach.

#### Downstream analysis

Dimensionality reduction and clustering were performed by PCA and visualized with Uniform Manifold Approximation and Projection (UMAP). Cell type identity was assigned to each cluster based on significance in overlap between signature genes of BMMNCs^35^ and HSPCs^40^ and cluster-specific genes (Fisher’s exact test). Palantir^73^ was used to reconstruct a differentiation continuum of cells and to order individual cells’ differentiation for pseudotime analysis. Gene Set Enrichment Analysis (GSEA; http://software.broadinstitute.org/gsea) and Gene Ontology (GO)^85^^,^^86^ were used to interpret gene set enrichment and pathways of defined differentially expressed genes. Single-nucleotide variations in *UBA1* and *DNMT3A* were identified in single cells using a Pysam-based tool, cb_sniffer with default parameters.^48^ A scoring algorithm to calculate interaction scores,^87^ CellPhoneDB^52^,^71^ and NicheNet5^1^ were used to examine ligand-receptor interactions.

### Quantification and statistical analysis

#### Unsupervised dimensionality reduction and UMAP visualization

PCA was used to reduce feature dimensions on the pooled cells of all patients and healthy donors, and top 30 principal components were input into t-SNE for further dimensional reduction. We found that cells of individuals clustered together, due to subject specificity and batch effects. The canonical correlation analysis (CCA) algorithm^83^ implemented in Seurat is a multivariate statistical technique for detecting the statistically common factors among digital gene expression (DGE) matrices, which varies from each other due to batch effects. After alignment with CCA, cells from different subjects were mixed well and separated by cell type categories. Resolution in the FindClusters function in Seurat^88^ was set to 2 for BMMNCs and 1 for HSPCs, and clustering results were shown in PCA and UMAP plots. Accordingly, marker genes in each cluster were identified using the Wilcoxon Rank-Sum test implemented in the Seurat v.2.3.4 package.

#### Cell type assignment

For lineage^−^CD34^+^ cells, an HSPC type was assigned to each cluster based on significance in overlapping between HSPCs and cluster-specific genes (the Fisher’s exact test).^28^^,^^40^ More specifically, top 250 overexpressed genes in each HSPC population were downloaded from http://www.jdstemcellresearch.ca/node/32, and were denominated as cell-type specific signature genes. Subsequently, the one-tailed Fisher’s exact test was utilized to assert enrichment of HSPC signature genes in the cluster marker gene list for each cluster,^69^ and a top associated cell type was assigned to each cluster. Cell types of BM cells were assigned with the same strategies using the Human Cell Atlas as ref. ^35^

#### Single-cell mutation identification and analysis

Aligned sequence data were generated by cellranger, and single-nucleotide variations in *UBA1* and *DNMT3A* were identified in single cells using the Pysam-based tool, cb_sniffer (https://github.com/sridnona/cb_sniffer), with default parameters.^48^ Reads that had no Chromium Cellular Barcode (CB) tag or no Chromium Molecular Barcode (UB) tag were filtered out. Then, cell-associated tags for downstream analyses of UMIs were obtained. Usually, duplicate reads existed for a given UB and a base at a mutant position were identical across all reads. In rare cases when there were inconsistent reads, the most common base was chosen if a mutation was present in at least 75% of the reads. All reads corresponding to the UB were discarded when there was no common base at a mutation position (>75% reads).

#### Reconstruction of hematopoiesis trajectories using scRNA-seq data and dynamic gene expression

We used Palantir,^73^ a recently published trajectory-detection algorithm for pseudotime ordering. Palantir is based on results of diffusion maps, which is suitable to explore a differentiation trajectory. It firstly uses diffusion maps to focus on developmental trends and avoid spurious edges resulting from the sparsity and noise in scRNA-seq. Projecting the data onto top diffusion components effectively focuses edges in directions with high cell densities and reweighs similarity along these directions. Then, Palantir estimates probability of a cell in an intermediate state to reach any of terminal states of differentiation. It thus provides a quantitative measure of differentiation potential, in which multipotent cells have the highest differentiation potential and mature terminal states have the lowest potential. A high resolution achieved by Palantir allows detailed mapping of gene expression trends and dynamics that correlate with changes in lineage potential. After calculating diffusion components by using the Harmony augmented affinity matrix, Palantir orders cells along pseudotime that recapitulates known marker trends in development. Tracking gene expression changes along pseudotime enable determination of the differentiation change for each of the terminal fates. We used Palantir to estimate the differentiation status in order to characterize cells in patients with *UBA1* mutations.

#### Projection of patients’ cells to the map of normal hematopoiesis

To characterize early hematopoiesis in VEXAS patents, individual cells were projected onto the map of normal hematopoietic differentiation based on cell-by-cell comparison of the patterns of global gene expression and localization to the most similar healthy donor cells (by Pearson correlation). This strategy was used for mapping patients’ cells on t-SNE and diffusion map plots, locating pseudotime estimation for Palantir.

#### Comparison of lineage gene Area Under the Receiver Operating Characteristic Curve (AUC) scores

We calculated AUC scores of lineage-specific gene expression (HSC, MEP, GMP, and LymP) of single cells in individual patients, and average AUC scores^75^ of specific lineages of all cells in each patient were compared. Comparison between two groups was performed using Prism (v.7.02; the GraphPad Software), and results were shown as mean ± standard derivation. Statistical analysis was performed using the two-sided unpaired Mann-Whitney test for two groups. p < 0.05 was considered statistically significant.

#### Differential expression of genes and generation of heatmaps

Differentially expressed genes were defined with the FindMarkers function in Seurat, by comparing gene expression in one cell subset with expression in all others. Genes with p value <0.05 and Log2(average fold change) > 0.1 were regarded as differentially expressed genes. Heatmaps and network visualization were generated with ggplot2 and heatmap2 in the R package.

#### GO and pathway analysis

GO was assessed with the R package topGO v2.26 using the algorithm elim,^74^ a minimum node size of 10, and genes that were expressed over 100 cells as the background gene list. p values derived from the GO analysis were not corrected for multiple testing. We examined the biological processes GO terms (The Gene Ontology Consortium, 2019) and the KEGG pathways.^89^^,^^90^

GSEA is the widely used pathway analysis tool that determines whether pre-defined gene sets show statistically significant, concordant differences between two biological states. GSEA is based on fold changes of all detected genes. To create gene sets for a genome with custom annotations, we associated our genes with known KEGG pathways and manually created gene sets. Fgsea^51^ was used for GSEA and to plot the running normalized enrichment scores along the ranked gene list.

#### Inflammatory gene pathway activity score analysis

To compare inflammation in HSPCs of VEXAS with several other hematopoietic diseases with overlapping clinical features, we calculated activity scores (expression levels) of several inflammatory response pathways in HSPCs of VEXAS patients in the current study, with data from published datasets (E-MTAB_8884 for chronic myelomonocytic leukemia (CMML) patients, GSE137429 for myelodysplastic syndrome (MDS) patients, and GSE76312 for chronic myelogenous leukemia (CML) patients). Briefly, we downloaded the fastq files from ArrayExpress with access number E-MTAB_8884, and used Cellranger 2.0 to analyze gene expression for this dataset. We also downloaded the processed scRNA-seq data for MDS (GSE137429) and CML (GSE76312) patients. We then downloaded the gene lists of HALLMARK_INFLAMMATORY_RESPONSE, HALLMARK_TNFA_SIGNALING VIA NFKB, and HALLMARK_INTERFERON_GAMMA_RESPONSE from MSigDB of GSEA, and their activity scores (expression levels) in our and these three datasets were calculated with the addModuleScore function built in the Seurat (http://satijalab.org/seurat/). The activity scores were normalized with healthy donors included in individual studies, and the double-sided t-test was used to assess the difference between VEXAS and three other diseases (MDS, CML, and CMML).

#### Inflammatory and cytokine score calculation

Inflammatory and cytokine scores were defined based on the published reference gene list,^43^^,^^49^ and were evaluated with the AddModuleScore function built in Seurat.^70^

#### Cell cycle stages calculation

Cell cycle stages were assigned with the CellCycleScoring function in Seurat.^70^^,^^91^ In specific, it calculated the mean expression levels of 43 “S phase" marker genes and 54 “G2/M phase" marker genes to obtain standardized scores for S and G2/M phases for each cell, and then assigns each cell to a specific phase with the highest score.

#### Ligand receptor analysis

Cell-cell interactions based on the expression of known ligand-receptor pairs in different cell types were calculated using the CellPhone DB version 3.1.0.^52^,^71^ Sorted CD34^+^ HSPCs were merged with defined HSPCs in BMMNCs as one population, and the algorithm was run on log-normalized expression values for cell populations of BMMNCs with default parameters and no subsampling to identify the enriched ligand-receptor pairs in VEXAS patients and healthy controls.

To examine and quantify a ligand–receptor interaction between different cell types, we implemented the established scoring algorithm proposed by Kumar et al. (2018)^87^ to calculate an interaction score based on ligand and receptor expression abundance. First, we collected 1,141 curated ligand-receptor pairs from the KEGG database for analysis. Then, for each cell, the gene expression of ligand ($E_{L})$ and receptor ($E_{R})$ were normalized to ($E_{L}^{norm}$ and $E_{R}^{norm})$ by subtracting an average housekeeping expression value. A score of a given ligand-receptor interaction between cell types A and B was calculated as a product of average ligand expression across all cells of type A and average receptor expression across all cells of type B ($S_{L,R=}\bar{E_{L,A}^{norm}}\times \bar{E_{R,B}^{norm}})$. The one-sided Wilcoxon rank-sum test was applied on the hypothesis that the interaction score was greater than 0. For each HSPC, the interaction score was defined as the sum of its L-R scores with all monocytes/granulocytes in BMMNCs $(S=\sum_{L,R\in LR\;pairs}S_{L,R}$).

#### NicheNet analysis

We used the R package NicheNet^53^ to predict ligand-receptor interactions that might drive gene expression changes in our cell types of interest. We combined all HSPCs, monocytes, and neutrophils for this analysis. All default parameters were used with an exception of setting a lower cutoff threshold of 0.3 and 0.6 for “prepare_ligand_target_visualization”.

#### Diversity index calculation

There are many ways of defining the diversity of a population, clonal types in this study, with each method providing a different representation of the number of clones (identical TCR/BCR chains) present (richness) and of their relative frequency (evenness). The Shannon entropy weighs both of these aspects of diversity equally, and it is an intuitive measure whereby the maximum value is determined by a total size of the repertoire. Entropy values decreases with increasing inequality of frequency as a result of clonal expansion. The Shannon entropy in a population of N clones with nucleotide frequency pi is defined by the following equation:

$$H\left(P\right)=-\sum_{i=1}^{n}p_{i}\;\log_{2}p_{i}$$

The Gini coefficient is a number aimed at measuring the inequality in a distribution. It is most often used in economics to measure a country’s wealth distribution and has been widely used in diversity assessment of TCRs/BCRs.^55^ The Gini coefficient is usually defined mathematically based on the Lorenz curve or Relative mean absolute difference.^92^ The Gini index and Shannon entropy for diversity and clonality analysis were calculated with the R package of tCR (https://imminfo.github.io/tcr/).

#### Identification of TCR motifs with shard antigen specificity using GLIPH2

GLIPH2^93^ was applied to T cells of VEXAS patients to identify clusters of TCRs that recognized the same epitope based on CDR3β amino acid sequence similarities, with default parameters. CDR3β amino acid sequences of the top 1,000 most abundant CDRs were used to identify significant motif lists and associated TCR convergence groups. The motif-shared TCRs network was visualized using Cytoscape version 3.9.1.^94^

#### Statistical analysis

Pearson correlations between interaction scores and inflammatory and cytokine scores were calculated with the R package. Comparison between groups was performed using the GraphPad Prism (v.9.5.1; GraphPad software, La Jolla, CA), and results were shown as mean ± standard derivation.

### Additional resources

Analysis and visualization of the scRNA-seq datasets in this study can be performed at the interactive website https://shouguog.shinyapps.io/vexas_cd34_bm/. DOIs are listed in the key resources table.

## Data Availability

The raw and analyzed sequencing data in this study have been deposited in the NCBI’s Gene Expression Omnibus (Database: GSE196052) and Sequence Read Archive (Database: SRP358093), and are publicly available. Accession numbers are listed in the key resources table. Code supporting this study is available at a dedicated Github repository [https://github.com/shouguog/UBA1]. Analysis and visualization of the scRNA-seq datasets in this study can be performed at the interactive website https://shouguog.shinyapps.io/vexas_cd34_bm/. DOIs are listed in the key resources table. All other relevant data supporting the key findings of this study are available within the article, and any additional information required to reanalyze the data reported in this paper is available from the lead contact upon request.

## Supplemental information

Document S1. Figures S1–S7

Table S1. Clinical and laboratory characteristics of patients with VEXAS syndrome, related to Figure 1A

Table S2. UBA1 mutations in BMMNCs and HSPCs, and gene set enrichment analysis results of differentially expressed genes in UBA1-mutated cells, related to Figure 3

Table S3. Differentially expressed genes and gene set enrichment analysis results in four cell lines with UBA1 knockdown, related to Figure 3F

Table S4. DNMT3A mutations in BMMNCs and HSPCs, and gene set enrichment analysis results of differentially expressed genes in DNMT3A-mutated cells, related to Figure S4

Table S5. Single-cell RNA sequencing metrics summary, related to STAR Methods Cell preparation, whole transcriptome amplification (WTA), cDNA library preparation, and sequencing

Document S2. Article plus supplemental information
