# Supplementary material for: Early activation of inflammatory pathways in UBA1-mutated hematopoietic stem and progenitor cells in VEXAS
Source: Cell Rep Med. 2023 Aug 15;4(8):101160. doi: 10.1016/j.xcrm.2023.101160 (PMC10439277; doi:10.1016/j.xcrm.2023.101160)
Supplement: Document S1. Figures S1–S7 [file mmc1.pdf]

Cell Reports Medicine, Volume *4*

Supplemental information

Early activation of inflammatory pathways in *UBA1*-mutated hematopoietic stem and progenitor cells in VEXAS

Zhijie Wu, Shouguo Gao, Qingyan Gao, Bhavisha A. Patel, Emma M. Groarke, Xingmin Feng, Ash Lee Manley, Haoran Li, Daniela Ospina Cardona, Sachiko Kajigaya, Lemlem Alemu, Diego Quinones Raffo, Amanda K. Ombrello, Marcela A. Ferrada, Peter C. Grayson, Katherine R. Calvo, Daniel L. Kastner, David B. Beck, and Neal S. Young

SUPPLEMENTAL INFORMATION

Early activation of inflammatory pathways in *UBA1*-mutated hematopoietic stem and progenitor cells in VEXAS

Short title: single-cell transcriptome in VEXAS syndrome

Zhijie Wu,[1,7,9*] Shouguo Gao,[1,7] Qingyan Gao,[1] Bhavisha A. Patel,[1] Emma M. Groarke,[1] Xingmin Feng,[1] Ash Lee Manley,[1] Haoran Li,[1] Daniela Ospina Cardona,[2,5,6] Sachiko Kajigaya,[1] Lemlem Alemu,[1] Diego Quinones Raffo,[1] Amanda K. Ombrello,[2] Marcela A. Ferrada,[3] Peter C. Grayson,[3] Katherine R. Calvo,[4] Daniel L. Kastner,[2] David B. Beck,[2,5,6,8*] and Neal S. Young[1,8]

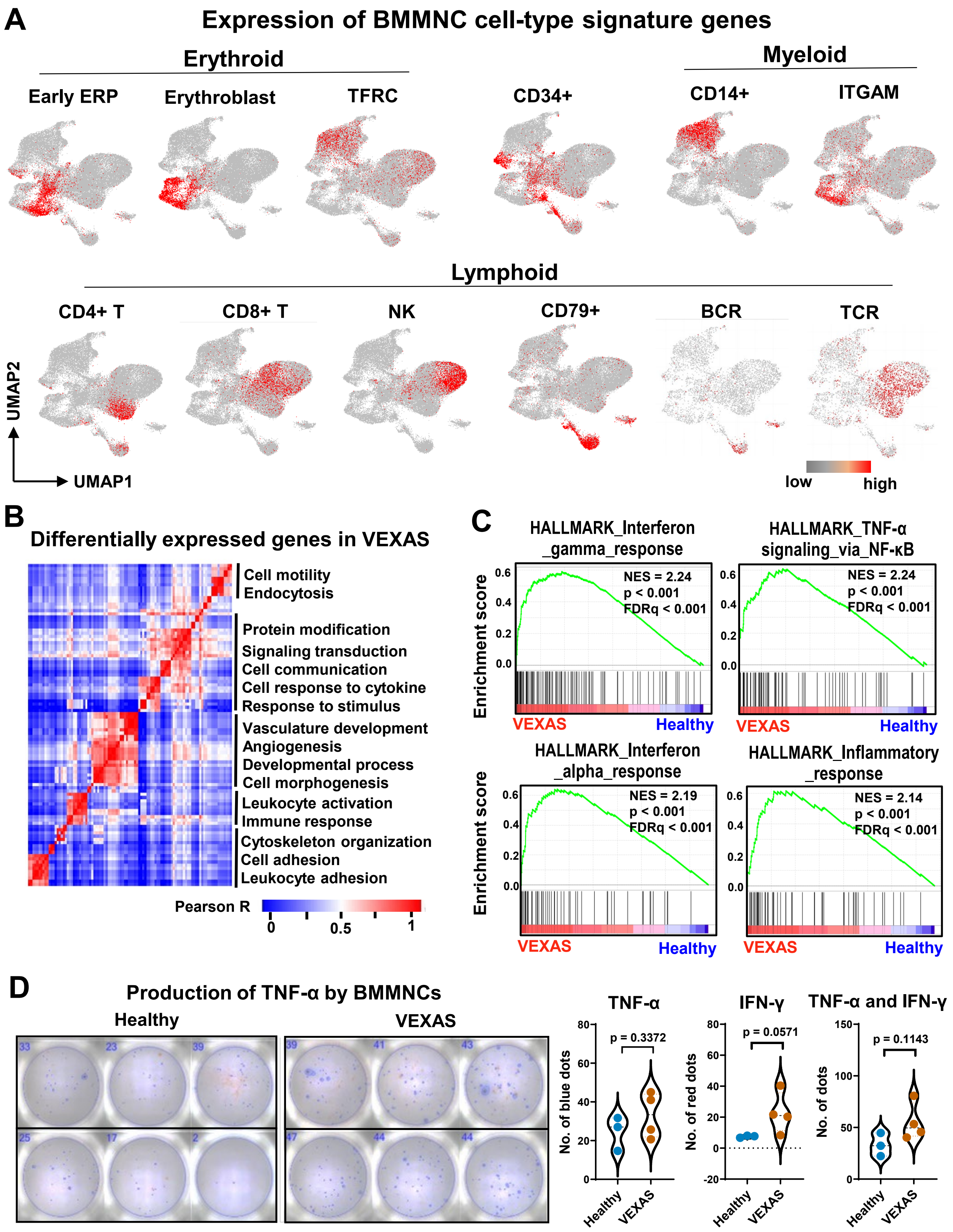

Figure S1. A distinct transcriptional and immunogenic profile of BMMNCs in VEXAS. Related to Figure 1.

(A) Expression of lineage signature genes[1] or single cell-type specific genes are highlighted in Uniform Manifold Approximation and Projection (UMAP) plots of batch-corrected single-cell gene expression in BMMNCs of all VEXAS patients and healthy donors: the same UMAP plots in Figure 1B.

(B) Gene-ontology (GO) semantic similarity matrix of differentially expressed genes in VEXAS. GO terms involved in similar functional matrices were adjacent and formed a block with Pearson R values ranging from 0 to 1. Terms noted on the right side depict common biological processes from blocks of GO terms.

(C) Gene Set Enrichment Analysis (GESA) plots of expressed genes of BMMNCs in VEXAS patients compared with those in healthy donors. GSEA enrichment plots for represented signaling pathways upregulated in HSPCs in VEXAS patients. GSEA were based on the Kolmogorov Smirnov test.

(D) Representative ELISpot wells showing IFN-γ secretion by BMMNCs from two VEXAS patients and two healthy donors in a first batch of a validation cohort, in triplicate. Right, quantification of IFN-γ or TNF-α-positive spots in BMMNCs plated (VEXAS patients n = 5 and healthy donors n = 2, in triplicate). p-values with the two-sided unpaired Mann-Whitney test are shown.

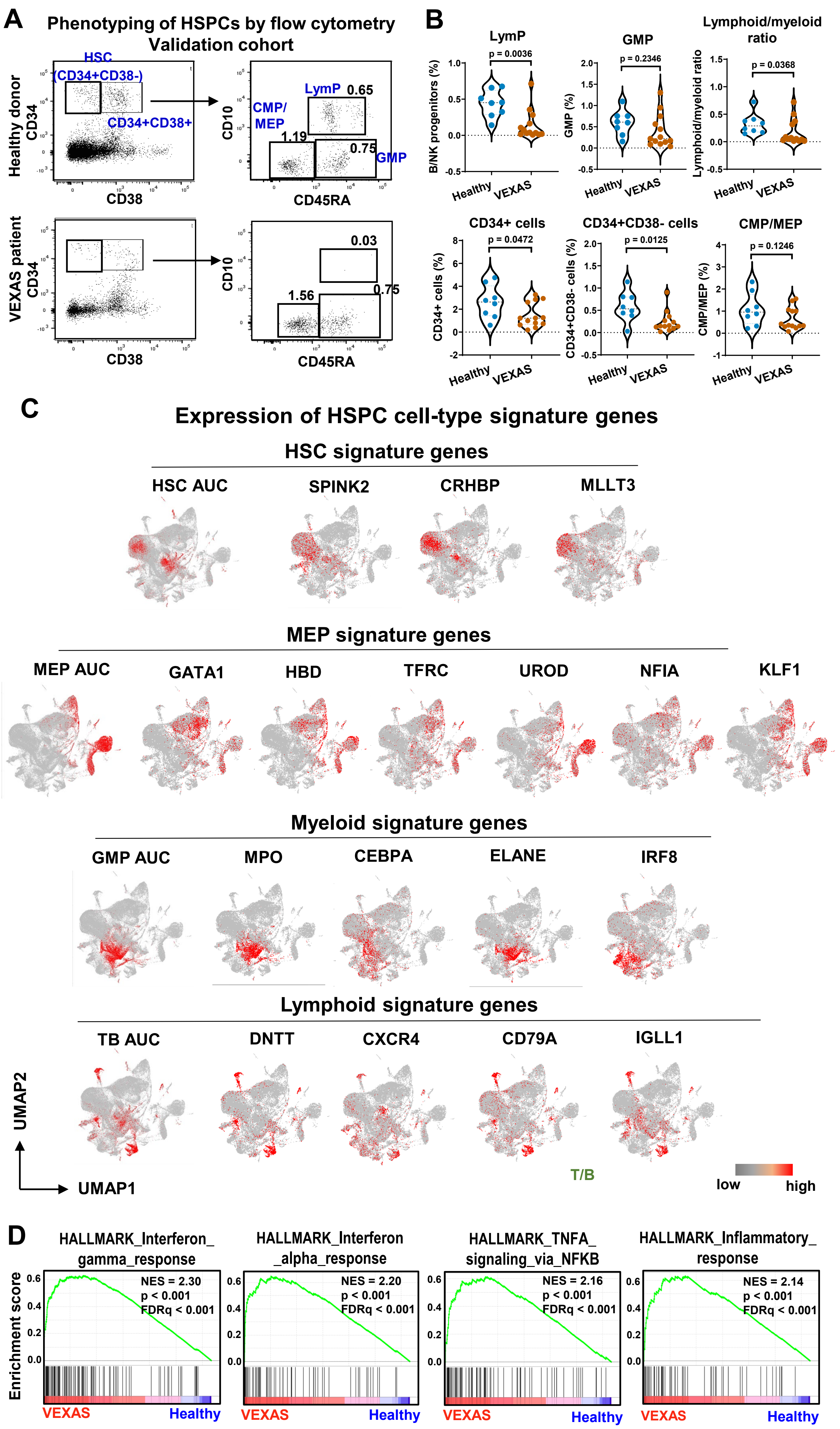

A
Phenotyping of HSPCs by flow cytometry
Validation cohort
HSC (CD34+CD38-)
CD34+CD38+
Healthy donor CD34
CD38
LymP 0.65
CMP/MEP
1.19
0.75
GMP
CD10
CD45RA
VEXAS patient CD34
0.03
1.56
0.75
B
LymP
p = 0.0036
B/NK progenitors (%)
GMP
p = 0.2346
GMP (%)
Lymphoid/myeloid ratio
p = 0.0368
CD34+ cells
p = 0.0472
CD34+ cells (%)
CD34+CD38- cells
p = 0.0125
CD34+CD38- cells (%)
CMP/MEP
p = 0.1246
CMP/MEP (%)
Healthy
VEXAS
C
Expression of HSPC cell-type signature genes
HSC signature genes
HSC AUC
SPINK2
CRHBP
MLLT3
MEP signature genes
MEP AUC
GATA1
HBD
TFRC
UROD
NFIA
KLF1
Myeloid signature genes
GMP AUC
MPO
CEBPA
ELANE
IRF8
Lymphoid signature genes
TB AUC
DNTT
CXCR4
CD79A
IGLL1
UMAP2
UMAP1
T/B
low
high
D
HALLMARK_Interferon_gamma_response
NES = 2.30
p < 0.001
FDRq < 0.001
HALLMARK_Interferon_alpha_response
NES = 2.20
p < 0.001
FDRq < 0.001
HALLMARK_TNFA_signaling_via_NFKB
NES = 2.16
p < 0.001
FDRq < 0.001
HALLMARK_Inflammatory_response
NES = 2.14
p < 0.001
FDRq < 0.001
Enrichment score
VEXAS
Healthy

Figure S2. Distinct transcriptional and phenotypic profiles of HSPCs in VEXAS. Related to Figure 2.

(A) Phenotypes of HSPCs in VEXAS patients and healthy donors by flow cytometry. Cell populations were defined as reported:[2] HSC, Lineage$^-$CD34$^+$CD38$^-$; CMP/MEP, Lineage$^-$CD34$^+$CD38$^+$CD10$^-$CD45RA$^-$; GMP, Lineage$^-$CD34$^+$CD38$^+$CD10$^-$CD45RA$^+$; LymP, Lineage$^-$CD34$^+$CD38$^+$CD10$^+$. HSC, hematopoietic stem cells and multipotent progenitors; CMP, multipotent common myeloid progenitor; MEP, megakaryocytic-erythrocytic progenitors; GMP, granulocytic-monocytic progenitors; LymP, lymphoid progenitors.

(B) Proportions of progenitor populations quantified by flow cytometry in (E) were compared between the validation cohort of VEXAS patients (n = 11) and healthy donors (n = 8). Data are shown with mean values ± standard error of the mean (SEM). p-values with the two-sided unpaired Mann-Whitney test are shown.

(C) Expression of lineage signature genes[3] or single cell-type specific genes are highlighted in UMAP plots of batch-corrected single-cell gene expression in BMMNCs of all VEXAS patients and healthy donors: the same UMAP plot in Figure 2C.

(D) GESA plots of differentially expressed genes of HSPCs in VEXAS patients compared with those in healthy donors, showing GSEA enrichment plots for represented signaling pathways upregulated in HSPCs in VEXAS patients. GSEA was based on the Kolmogorov Smirnov test.

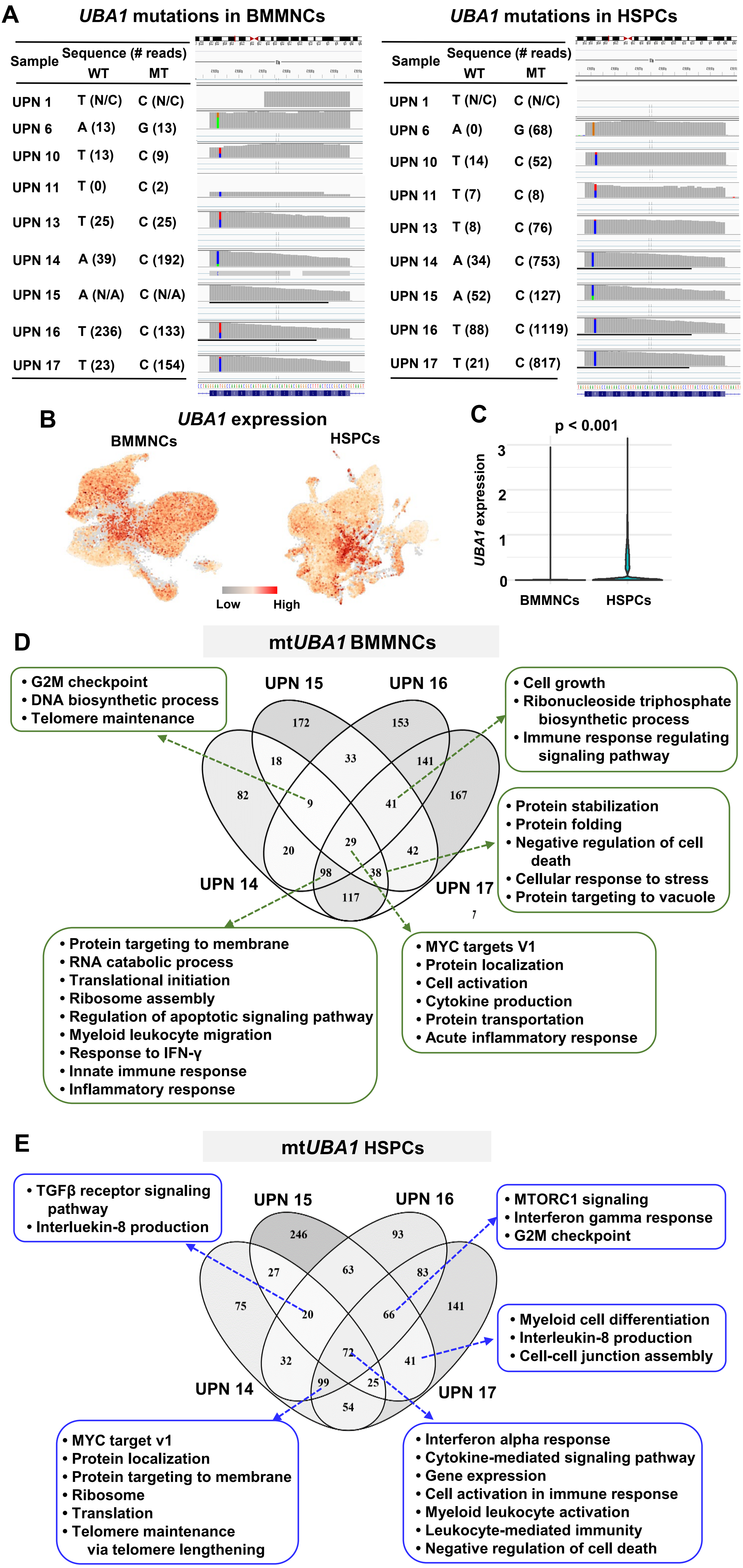
A
UBA1 mutations in BMMNCs
Sample
Sequence (# reads)
WT
MT
UPN 1 T (N/C) C (N/C)
UPN 6 A (13) G (13)
UPN 10 T (13) C (9)
UPN 11 T (0) C (2)
UPN 13 T (25) C (25)
UPN 14 A (39) C (192)
UPN 15 A (N/A) C (N/A)
UPN 16 T (236) C (133)
UPN 17 T (23) C (154)
UBA1 mutations in HSPCs
Sample
Sequence (# reads)
WT
MT
UPN 1 T (N/C) C (N/C)
UPN 6 A (0) G (68)
UPN 10 T (14) C (52)
UPN 11 T (7) C (8)
UPN 13 T (8) C (76)
UPN 14 A (34) C (753)
UPN 15 A (52) C (127)
UPN 16 T (88) C (1119)
UPN 17 T (21) C (817)
B
UBA1 expression
BMMNCs
HSPCs
Low
High
C
p < 0.001
UBA1 expression
3
2
1
0
BMMNCs
HSPCs
D
mtUBA1 BMMNCs
UPN 15
UPN 16
UPN 14
UPN 17
172
153
18
33
141
82
9
41
167
20
29
42
98
38
117
• G2M checkpoint
• DNA biosynthetic process
• Telomere maintenance
• Cell growth
• Ribonucleoside triphosphate biosynthetic process
• Immune response regulating signaling pathway
• Protein stabilization
• Protein folding
• Negative regulation of cell death
• Cellular response to stress
• Protein targeting to vacuole
• Protein targeting to membrane
• RNA catabolic process
• Translational initiation
• Ribosome assembly
• Regulation of apoptotic signaling pathway
• Myeloid leukocyte migration
• Response to IFN-γ
• Innate immune response
• Inflammatory response
• MYC targets V1
• Protein localization
• Cell activation
• Cytokine production
• Protein transportation
• Acute inflammatory response
E
mtUBA1 HSPCs
UPN 15
UPN 16
UPN 14
UPN 17
246
93
27
63
83
75
20
66
141
32
72
41
99
25
54
• TGFβ receptor signaling pathway
• Interleukin-8 production
• MTORC1 signaling
• Interferon gamma response
• G2M checkpoint
• Myeloid cell differentiation
• Interleukin-8 production
• Cell-cell junction assembly
• MYC target v1
• Protein localization
• Protein targeting to membrane
• Ribosome
• Translation
• Telomere maintenance via telomere lengthening
• Interferon alpha response
• Cytokine-mediated signaling pathway
• Gene expression
• Cell activation in immune response
• Myeloid leukocyte activation
• Leukocyte-mediated immunity
• Negative regulation of cell death

Figure S3. Expressed *UBA1* mutations in VEXAS detected by scRNA-seq. Related to Figure 3.

(A) Expressed *UBA1* mutations in BMMNCs (left) and HSPCs (right) in UPNs14-17 detected by scRNA-seq. Sequences and numbers of wild-type and mutant reads in individual samples are indicated on the left. Figures of mutations using the Integrative Genomics Viewer (IGV from the Broad Institute) are shown on the right.

(B) UMAP plots of single-cell gene expression in BMMNCs and HSPCs of all healthy donors, colored with *UBA1* expression levels.

(C) Violin plots showed *UBA1* expression levels in BMMNCs were significantly higher than those in HSPCs in healthy donors. The two-sided unpaired t-test. p-value < 0.001.

(D) In individual VEXAS patients, gene expression was compared between myeloid BMMNCs with mutant *UBA1* (mt*UBA1*) and those with wild-type *UBA1* (wt*UBA1*). A Venn diagram shows upregulated pathways of differentially expressed genes in at least three patients (among UPNs 14-17).

(E) Gene expression of mt*UBA1* HSPCs was compared to that of wt*UBA1* HSPCs in individual VEXAS patients. A Venn diagram shows upregulated pathways of differentially expressed genes in at least three patients (among UPNs 14-17).

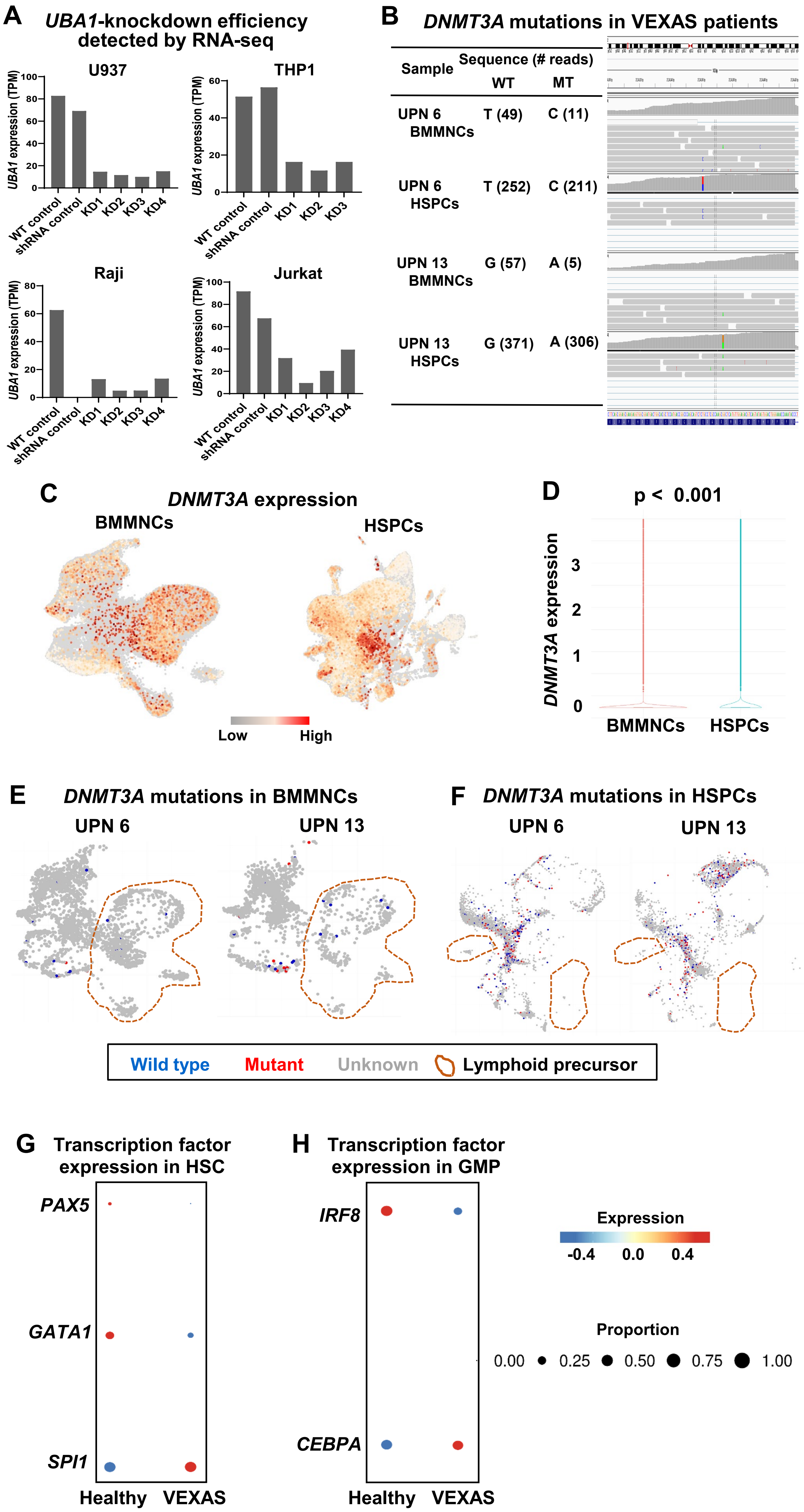
A
UBA1-knockdown efficiency detected by RNA-seq
U937
THP1
Raji
Jurkat
UBA1 expression (TPM)
WT control
shRNA control
KD1
KD2
KD3
KD4
B
DNMT3A mutations in VEXAS patients
Sample
Sequence (# reads)
WT
MT
UPN 6 BMMNCs
T (49)
C (11)
UPN 6 HSPCs
T (252)
C (211)
UPN 13 BMMNCs
G (57)
A (5)
UPN 13 HSPCs
G (371)
A (306)
C
DNMT3A expression
BMMNCs
HSPCs
Low
High
D
p < 0.001
DNMT3A expression
BMMNCs
HSPCs
E
DNMT3A mutations in BMMNCs
UPN 6
UPN 13
F
DNMT3A mutations in HSPCs
UPN 6
UPN 13
Wild type
Mutant
Unknown
Lymphoid precursor
G
Transcription factor expression in HSC
PAX5
GATA1
SPI1
Healthy
VEXAS
H
Transcription factor expression in GMP
IRF8
CEBPA
Healthy
VEXAS
Expression
-0.4
0.0
0.4
Proportion
0.00
0.25
0.50
0.75
1.00

Figure S4. Expressed *DNMT3A* mutations in VEXAS detected by scRNA-seq. Related to Figure 3.

(A) Knockdown efficiency of *UBA1* in four cell lines (U937, THP1, Raji, and Jurkat) detected by RNA-seq analysis.

(B) *DNMT3A* mutations in BMMNCs and HSPCs in UPN6 and UPN13 detected by scRNA-seq analysis, respectively. Sequences and numbers of wild-type and mutant reads in individual samples are indicated on the left. Figures of mutations using the Integrative Genomics Viewer (IGV) are shown on the right.

(C) UMAP plots of single-cell gene expression in BMMNCs and HSPCs of all healthy donors, colored by *DNMT3A* expression levels.

(D) Violin plots show *DNMT3A* expression levels in BMMNCs were higher in HSPCs than in healthy donors. Data were analyzed with the two-sided unpaired t-test. p-value < 0.001.

(E) UMAP plots of single-cell gene expression in BMMNCs of VEXAS patients, the same t-SNE plot as Figure 1B left. Cells with expressing mutant *DNMT3A* (mt*DNMT3A*) or wild-type *DNMT3A* (wt*DNMT3A*) are colored as red or blue dots, respectively, and all the other cells as grey. Lymphoid precursors are circled on t-SNE plots.

(F) UMAP plots of single-cell gene expression in BMMNCs of VEXAS patients, the same t-SNE plot as Figure 2C. Cells with expressing mt*DNMT3A* or wt*DNMT3A* are colored as red or blue dots, respectively, and all the other cells as grey.

(G) A bubble plot showing expression levels of transcription factor genes *PAX5*, *GATA1*, and *SPI1* in HSCs of VEXAS patients, compared with those in healthy donors.

(H) A bubble plot showing expression levels of transcription factor genes *IRF8* and *CEBPA* in GMP of VEXAS patients, compared with those in healthy donors.

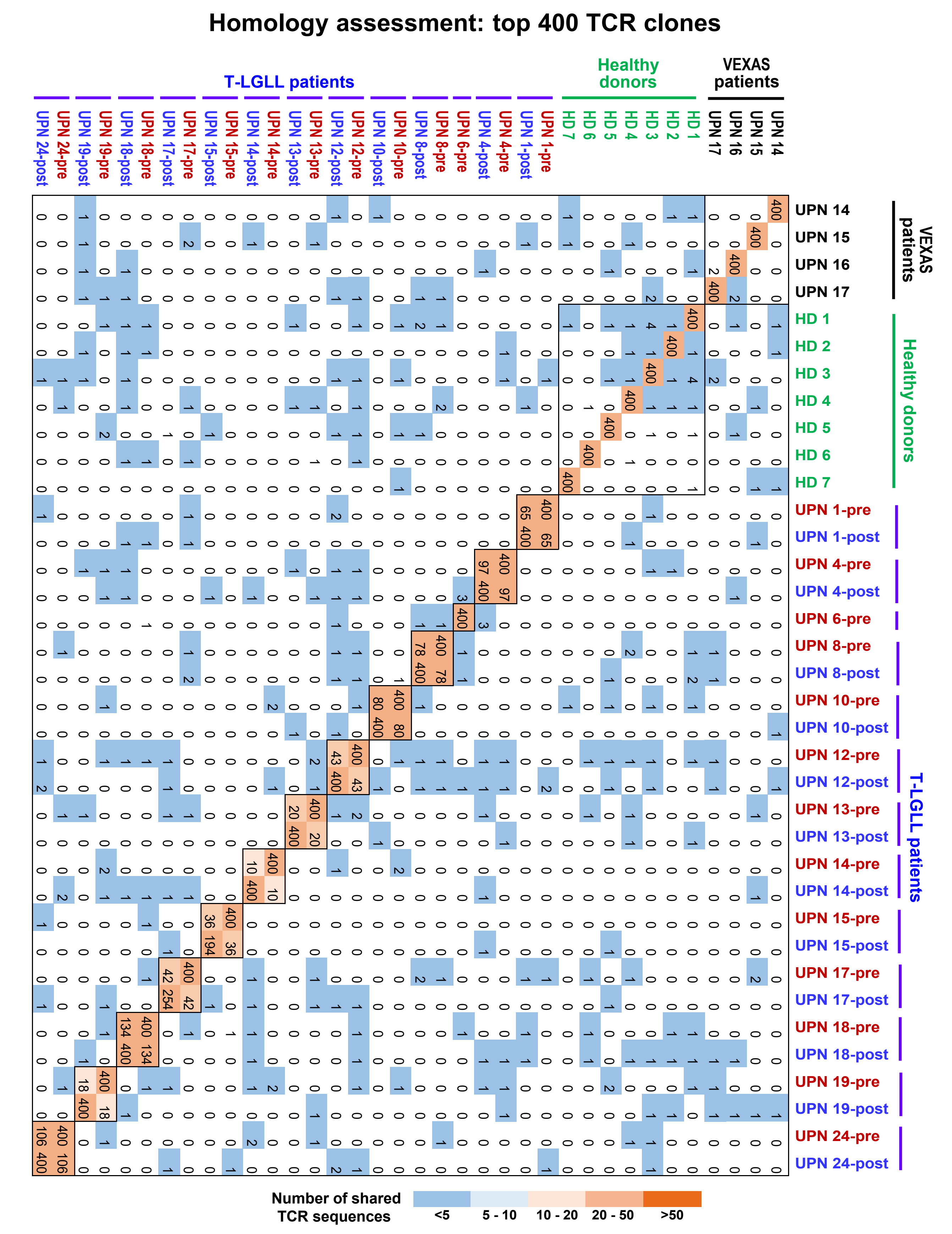

Figure S5. A lack of common TCR clonotypes in VEXAS patients. Related to Figure 7.

A heatmap plot showing the number of common TCR clones in UPNs 14-17, healthy donors (n = 7), and serial samples of T-LGLL patients (n = 13)[4] among top 400 TCR clones. Both x- and y-axes represent samples of patients and healthy donors. Paired samples of the same T-LGLL patient were adjacent. Numbers indicate counts of identical TCR clones shared among samples. A color scheme ranging from dark orange to dark blue represents the number of shared CDR sequences from high to low. In general, there was lack of common TCR usage in VEXAs patients (UPNs 14-17), and few common TCR clones in healthy individuals or T-LGLL patients. There was also a lack of common TCR usage among healthy individuals or among T-LGLL patients. HD, healthy donor; T-LGLL, T large granular lymphocytic leukemia; UPN, unique patient number.

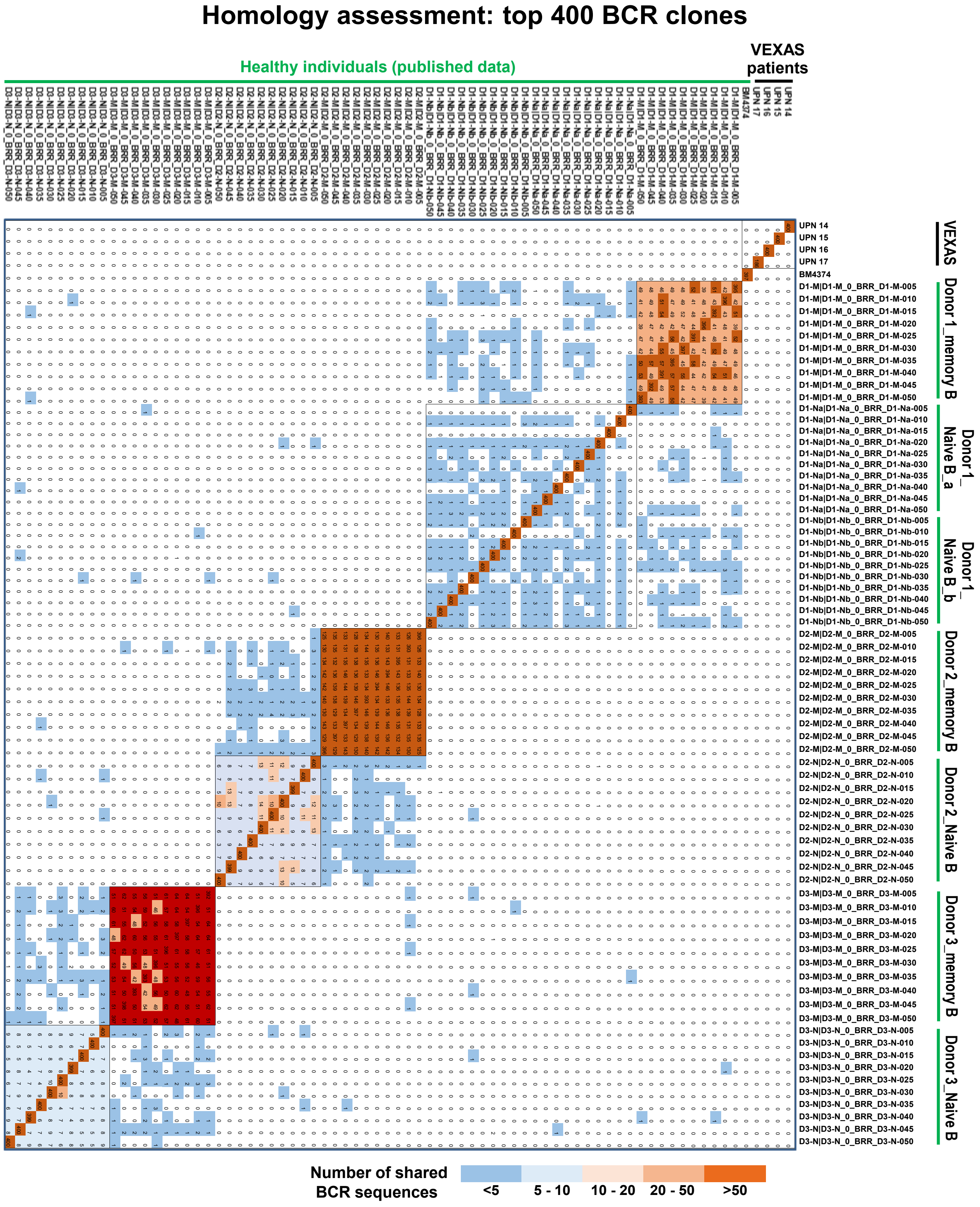

Figure S6. A lack of common BCR clonotypes in VEXAS patients. Related to Figure 7.

A heatmap plot showing the number of common BCR clones in UPNs 14-17, and duplicated memory B cell and naïve B cell samples from healthy donors (n = 3)[5] among top 400 TCR clones. Both x- and y-axes show samples of patients and healthy donors. Samples of the same healthy individual were adjacent. Numbers indicate counts of identical BCR clones shared among samples. A color scheme ranging from dark orange to dark blue represents the number of shared IgH sequences from high to low. In general, there was a lack of common BCR usage in VEXAs patients (UPNs 14-17), and no common BCR clones in these healthy individuals. There was also a lack of common BCR usage among healthy individuals. HD, healthy donor; UPN, unique patient number.

A

BCR clonality and expressed *UBA1* mutations in VEXAS

	UPN 14	UPN 15	UPN 16	UPN 17
Total cell number with detected BCR	1048	575	1219	339
No. 1 clone size	62	186	449	317
No. 2 clone size	49	42	6	76
No. 3 clone size	33	5	2	52
No. 4 clone size	28	4	1	33
Medium clone size (25 -75 percentile)	12 (6-24)	4 (2-42)	4 (1.25-338.3)	21 (3.25-47.25)
mt*UBA1* single cells	7	1	9	2
Clone size (CDR3)	12 CARDLRWELGEGGFDPW<br>8 CALRRQYDLSENRGSGWFDPW<br>5 CVRIYYGNRNFHRFDAFDIW<br>2 CSCEELW<br>2 CARPATTNAYYYYYYMDVW<br>1 CARDRSRGAKAPTAYIDHW<br>1 CAKDRGPVVGSRGCDFW	186 CARNLLMWFGEFYPW	449 CAKVYSGEMATMFGFDHSHYYGMDVW<br>449 CAKVYSGEMATMFGFDHSHYYGMDVW<br>449 CAKVYSGEMATMFGFDHSHYYGMDVW<br>449 CAKVYSGEMATMFGFDHSHYYGMDVW<br>449 CAKVYSGEMATMFGFDHSHYYGMDVW<br>7 CAKRTGGNNGPFDYW<br>1 CARGCSSVPCVW<br>1 CARDLVRIWNYVGVLDLW<br>1 CAKGDYDTRINTFQNW	31 CATTRLAQETYRVLELNWFDPW<br>1 CTRTTTVESAVFDYW
wt*UBA1* single cells	4	35	39	0
Clone size (CDR3)	5 CVRIYYGNRNFHRFDAFDIW<br>3 CASAPLSDDFWSHYYPGGMDVW<br>2 CAKDRANFYGPGIIDFW<br>1 CAAWGETAVRYHAFDIW	186 CARNLLMWFGEFYPW<br>186 CARNLLMWFGEFYPW<br>186 CARNLLMWFGEFYPW<br>186 CARNLLMWFGEFYPW<br>42 CARHDNTGSYCLFYW<br>2 CVTSWFYGSGYVYFHQW<br>2 CSRHSMRAPEFFDFW<br>2 CARRLLYEGGTFDIW<br>2 CVTSWFYGSGYVYFHQW<br>1 CARERYCVGGIWCYYGMDVW<br>1 CAKSRTYYDFWSGYFDYW<br>1 CARHGVGATTDYYFDHW<br>1 CAKDFRESGDYGWYFDLW<br>1 CAYGTTVTTPFDSW<br>1 CAKFDVDCSGGACQSKVLYYFDNW<br>1 CARWVRTTTSRTFDYW<br>1 CVSHRAETGPFDYW<br>1 CARLEMGSIRHDAFDIW<br>1 CGRDGYFEAIEHW<br>1 CALSSDWVWYFDYW<br>1 CAKLHGSDYLPRFDPW<br>1 CAKDQFSTGLFVGQLAGDW<br>1 CARHRSDSSGRKWYFDLW<br>1 CARDLQGGHWYFDLW<br>1 CARNAELVVFAMGMRFWLDPW<br>1 CARDSSAYNYVPYYSYYHGMDVW<br>1 CAKSPGQRSGKLEDW<br>1 CARSYNTGWNDGAFDFW<br>1 CAKEQTLYDNRVNNFDCW<br>1 CRGKGGEIDSW<br>1 CARDVAGVLDYW<br>1 CAKVLVPTAIFHAFDIW<br>1 CGRDSQGLGIPGYYVEHW<br>1 CARHGVGATTDYHFDLW<br>1 CARVEYYGSGMVFDNW	449 CAKVYSGEMATMFGFDHSHYYGMDVW<br>449 CAKVYSGEMATMFGFDHSHYYGMDVW<br>449 CAKVYSGEMATMFGFDHSHYYGMDVW<br>449 CAKVYSGEMATMFGFDHSHYYGMDVW<br>449 CAKVYSGEMATMFGFDHSHYYGMDVW<br>449 CAKVYSGEMATMFGFDHSHYYGMDVW<br>449 CAKVYSGEMATMFGFDHSHYYGMDVW<br>449 CAKVYSGEMATMFGFDHSHYYGMDVW<br>449 CAKVYSGEMATMFGFDHSHYYGMDVW<br>449 CAKVYSGEMATMFGFDHSHYYGMDVW<br>6 CARALISVSPCDYW<br>6 CARALISVSPCDYW<br>6 CARALISVSPCDYW<br>6 CARALISVSPCDYW<br>2 CARGHNDYPGRWFDPW<br>2 CARDNGDSDLVLAYW<br>2 CATGGEMATIFESFDYW<br>1 CVKDIGAWFGETMFDPW<br>1 CARDRKWEQLLGYFDYW<br>1 CARPPPHVVVLPIVIRDWYFDLW<br>1 CARDRWLGW<br>1 CARLPVAGNSLYYYGLDVW<br>1 CAKDYIRSTTNFLGLNGMDVW<br>1 CANKLAVAGTYQPFVSGYW<br>1 CARVLRRGSSWHSENCFDPW<br>1 CARVDVW<br>1 CTGILPAANWASATSNYW<br>1 CARARADTAKIRFDYW<br>1 CARGSRPGGVYNWFDPW<br>1 CARSSRDHQLVLFVNW<br>1 CARARISSDSTVGYW<br>1 CAKAGDNYGSGSYYFDYW<br>1 CARGHIAVPGPVPAFDYW<br>1 CARGAHDFWNGYSVPDYW<br>1 CVRDRGYQSFDYW<br>1 CGRVVAGAPLPAHIDFW<br>1 CARGLRTSRYFDLW<br>1 CAKDKRMTTTYFHYW<br>1 CARGPDWFDPW	

B

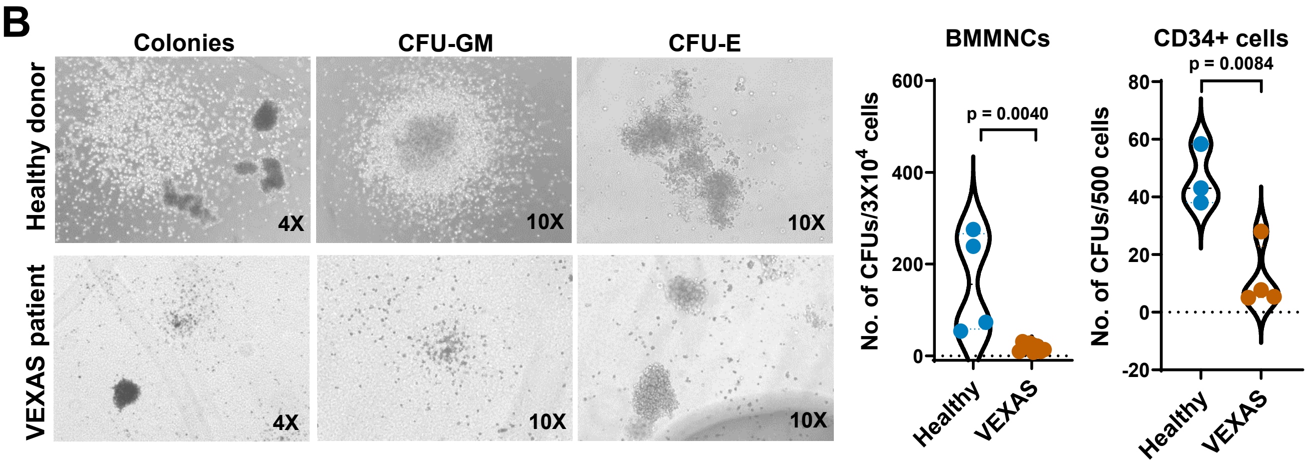

Figure S7. BCR clonality and expressed *UBA1* mutations in VEXAS detected by coupled scRNA-seq and scBCR-seq, and impaired differentiation and proliferation of hematopoietic progenitor cells in VEXAS. Related to Figure 7.

(A) BCR sequences of single-lineage^{-}CD34^{+} HSPCs with either mt*UBA1* or wt*UBA1* are described in UPNs 14-17. A total cell number with detected BCR expression, top 4 clone sizes (cell number), and medium clone sizes are shown on the top. The number of detected single mt*UBA1* and wt*UBA1* HSPCs in each individual, and sizes and sequences of the BCR clones they belonged to are shown at the bottom, respectively.

(B) Colony forming assays using BMMNCs and enriched CD34^{+}HSPCs in VEXAS patients and healthy donors. Representative images of colony forming units (CFUs) formed by BMMNCs or HSPCs, colony forming units for granulocytes and macrophages (CFU-GM), and colony forming units for erythrocytes (CFU-E) in patients and healthy donors are presented. The numbers of CFUs formed by BMMNCs (left) and CD34^{+}HSPCs (right) were compared. Data are presented as mean ± SEM. p-values calculated with the two-sided unpaired Mann-Whitney test are shown.

SUPPLEMENTAL REFERENCES

1. Hay, S.B., Ferchen, K., Chetal, K., Grimes, H.L., and Salomonis, N. (2018). The human cell atlas bone marrow single-cell interactive web portal. Exp. Hematol. *68*, 51-61.

2. Van Galen, P., Kreso, A., Mbong, N., Kent, D.G., Fitzmaurice, T., Chambers, J.E., Xie, S., Laurenti, E., Hermans, K., Eppert, K., et al. (2014). The unfolded protein response governs integrity of the haematopoietic stem cell pool during stress. Nature *510*, 268-272.

3. Laurenti, E., Doulatov, S., Zandi, S., Plumb, I., Chen, J., April, C., Fan, J.B., and Dick, J.E. (2013). The transcriptional architecture of early human hematopoiesis identifies multilevel control of lymphoid commitment. Nat. Immunol. *14*, 756-763.

4. Gao, S., Wu, Z., Arnold, B., Diamond, C., Batchu, S., Giudice, V., Alemu, L., Raffo, D.Q., Feng, X., Kajigaya, S., et al. (2022). Single-cell RNA sequencing coupled to TCR profiling of large granular lymphocyte leukemia T cells. Nat. Commun. *13*, 1982.

5. DeWitt, W.S., Lindau, P., Snyder, T.M., Sherwood, A.M., Vignali, M., Carlson, C.S., Greenberg, P.D., Duerkopp, N., Emerson, R.O., and Robins, H.S. (2016). A public database of memory and naïve B-cell receptor sequences. PLoS One. *11*, e0160853.
